# Cucurbitane Glycosides and Their Potential Anti-Inflammatory Activities from *Hemsleya chinensis* Tubers

**DOI:** 10.3390/molecules30112349

**Published:** 2025-05-28

**Authors:** Jun Chi, Miaomiao Li, Feihe Lian, Yixiao Li, Liping Dai

**Affiliations:** 1 Henan Collaborative Innovation Center for Research and Development on the Whole Industry Chain of Yu-Yao, Henan University of Chinese Medicine, Zhengzhou 450046, China; chijun16@126.com (J.C.); 1918290380@163.com (M.L.); 15738349001@163.com (F.L.); liyixiao0709@163.com (Y.L.); 2 Engineering Technology Research Center for Comprehensive Development and Utilization of Authentic Medicinal Materials in Henan Province, Henan University of Chinese Medicine, Zhengzhou 450046, China

**Keywords:** *Hemsleya chinensis* tubers, cucurbitane glycosides, cucurbitane triterpenes, anti-inflammatory, molecular docking

## Abstract

*Hemsleya chinensis* tubers, abundantly produced in southwestern China, are commonly used as a folk medicine that excel in anti-inflammation to treat enteritis, bronchitis, and tonsillitis. In this study, three previously undescribed cucurbitane glycosides, hemchinins G–H (**1**–**3**), that were characterized by the presence of four glucose substitutions, as well as eleven ones with one to three *β*-glucoses, were isolated from the tubers of *H. chinensis*. The structures were confirmed using comprehensive UV, IR, HR-ESI-MS, and NMR analyses, and absolute configurations were determined through a comparison of calculated and experimental ECD after acid hydrolysis. Compounds **1**–**3** showed NO inhibition effects on LPS-induced RAW 264.7 cells. Finally, molecular docking analyses were conducted to obtain the affinities of the isolated cucurbitane glycosides and our previously reported 19 cucurbitane triterpenes, focusing on targets involved in anti-inflammatory effects. The results indicated that they showed high docking scores of affinities with the proteins in the NF-κB, AMPK, and Nrf2 signaling pathways. Among them, cucurbitane triterpenes with sugar moiety substitution at C-3 and C-26/27 showed better affinity ability. The findings can provide insights into the anti-inflammatory mechanisms of this species and facilitate the development of novel therapeutic agents.

## 1. Introduction

*Hemsleya chinensis*, named “*Zhonghua Xuedan*” or “*Xuedan*” in Chinese, is a perennial climbing herb of the Cucurbitaceae family and is widely distributed in southwestern China, with abundant natural resources [1]. The tubers of *H. chinensis* are commonly used as a Chinese folk medicine in the provinces of Guizhou and Yunnan, with them excelling in clearing heat and detoxification, antisepsis, and anti-inflammation, to treat bacillary dysentery, enteritis, bronchitis, and tonsillitis [2,3]. Nowadays, it is documented in local standards of Chinese materia medica in the provinces of Guizhou (2019 Edition) [4] and Hubei (2018 Edition) [5]. The extract of *H. chinensis* tubers has been used as an important material for the preparation of *Xuedansu Tablets*, *Xuedan Gastrointestinal Pills*, *Xuedan Detoxification Pills*, etc., which show significant therapeutic effects in treating diseases such as gastric ulcers and pancreatitis [3]. In our previous research, we reported that the ethanolic extract of *H. chinensis* tubers can exert a protective effect on HCl/EtOH-induced acute gastric ulcers in rats, and its mechanism may be related to the inhibition of the expression of inflammatory mediators by the p38 MAPK/NF-κB signaling pathway [6]. The anti-inflammatory potential of *H. chinensis* tubers is derived from the combined effects of multiple active ingredients, which prompts us to systematically elucidate the material basis of these therapeutic effects.

Cucurbitane-type triterpenes represent a class of structurally diverse natural compounds that serve as key chemotaxonomic markers for the *Hemsleya* genus [7]. The cucurbitane skeleton is characterized by its highly oxygenated structures, typically featuring multiple oxygen-containing functional groups at positions C-3, C-11, and C-26/27. Some studies have proven that cucurbitane triterpenes exhibit antibacterial, anti-inflammatory, and anticancer actions, with them being recognized as the most active materials in *H. chinensis* [8]. Cucurbitane triterpenes cucurbicins IIa and IIb are the main components in *Xuedansu Tablets*, which have been clinically employed as an effective antibacterial and anti-inflammatory agent, particularly in the management of various digestive system disorders [9]. This study speculates that there is a series of novel and significantly active cucurbitane triterpenes in *H. chinensis* tubers. Based on the previous discovery of cucurbitane triterpenes in the EtOAc extraction of *H. chinensis* tubers [10], a special separation strategy for these components was developed, and a further exploration of structurally interesting and bioactive cucurbitane triterpenes from *n*-butanol and the remaining EtOAc fraction of this species was conducted.

**Figure 1:**
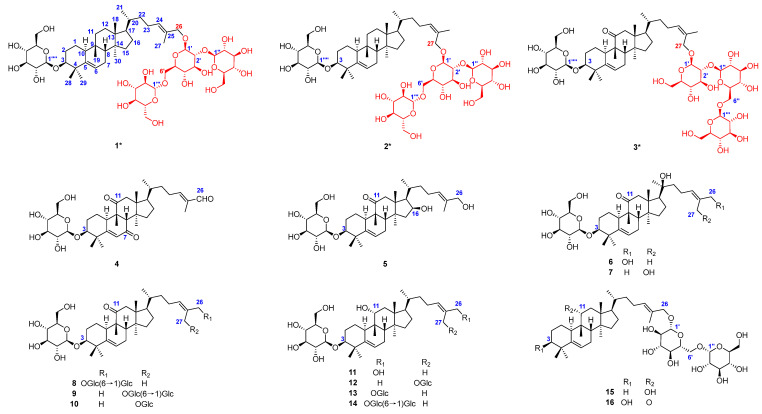
Chemical structure of the isolated compounds (* represented new compounds).

As a result, three previously undescribed cucurbitane glycosides containing four *β*-D-glucoses substitutions at positions C-3 and C-26/27, hemchinins G–I (**1**–**3**) (Figure 1), along with thirteen known ones (**4**–**16**) with one to three *β*-glucoses, including hemslepenside A (**4**) [11], xuedanoside H (**5**) [12], scandenoside R1 (**6**) [13], scandenoside R2 (**7**) [14], carnosifloside II (**8**) [15], scandenoside R5 (**9**) [13], 3-*O*-*β*-D-glucopyranosyl-3*β*,27-dihydroxycucurbita-5,24(*Z*)-diene-11 -one-27-*O*-*β*-D-glucopyranoside (**10**) [15], jinfushanoside G (**11**) [16], hemslepenside I (**12**) [17], hemslepenside H (**13**) [17], 3*β*,11*α*,26-trihydroxy-cucurbita-5,24(*E*)-diene-3-*O*-*β*-D-glucopyranosyl-26-*O*-*β*-D-glucopyranosyl (1→6)-*α*-L-arabinoside (**14**) [18], hemsleyaoside M (**15**) [19], and carnosifloside I (**16**) [15], were isolated and identified from the EtOAc and *n*-butanol fraction of *H. chinensis* tubers. The anti-inflammatory effects of compounds **1**–**3** were also evaluated on lipopolysaccharide (LPS)-induced RAW 264.7 macrophages. The findings contribute to our understanding of the bioactive components in *H. chinensis*.

Inflammation is a complex biological response that plays crucial roles in major human diseases such as gastrointestinal disease, cardiovascular diseases, and diabetes. Cucurbitane triterpenes have gained significant attention for their anti-inflammatory potential. The main pathways involved in inflammation include the following: The NF-κB pathway, which regulates the expression of pro-inflammatory genes such as COX-2 and iNOS [20]; the MAPK pathway, which controls the production of cytokines like TNF-α and IL-6 [21]; the PI3K/Akt pathway, which contributes to inflammation by regulating cell survival and NF-κB activation [22]; and the NLRP3 inflammasome pathway, which is critical for the maturation and secretion of IL-1*β* and IL-18 [23]. Other pathways, such as Nrf2/ARE [24], COX-2/PGE2 [25], and TGF-*β*/Smad [26], also play significant roles in modulating inflammatory responses through mechanisms involving lipid metabolism, oxidative stress, and tissue repair. Therefore, molecular docking analyses were performed to obtain the affinities of both the isolated cucurbitane glycosides and our previously reported 19 cucurbitane triterpenes [10], focusing on key targets involved in inflammatory pathways. The results indicated that they showed high docking scores of affinities with the proteins in the NF-κB, AMPK, and Nrf2 signaling pathways, and the presence of multiple glucose units at C-3 and/or C-26/27 positions and an acetyl group at C-25 were crucial for the potential bioactivity, which could provide insights into their anti-inflammatory mechanisms and facilitate the development of novel therapeutic agents.

## 2. Results and Discussion

### 2.1. Structural Elucidation of Isolated Compounds

Compound **1** was acquired as a white amorphous powder. Its HR-ESI-MS spectrum at *m/z* 1113.5814 [M + Na] ^+^ (calcd for C_54_H_90_O_22_Na, 1113.5816) and ^13^C NMR data established the molecular formula of C_54_H_90_O_22_. The observed IR absorptions were assigned as O-H stretching (3385 cm^−1^) of the hydroxy group, as well as C-O stretching (1038 cm^−1^) of the glycosidic bond (C1-O-C4) and primary alcohol. The ^1^H NMR (Table 1 and Table 2) and HSQC spectra of compound **1** exhibited the presence of two olefinic protons [*δ*_H_ 5.51 (m, H-6), 5.48 (m, H-24)], one oxymethylene proton [*δ*_H_ 4.21 (d, *J* = 11.7 Hz, H-26a), 4.01 (d, *J* = 11.7 Hz, H-26b)], one oxymethine proton [*δ*_H_ 3.40 (brs, H-3)], and seven methyl signals [*δ*_H_ 1.67 (s, H-27), 1.19 (s, H-28), 1.02 (s, H-29), 0.93 (d, *J* = 6.0 Hz, H-21), 0.89 (s, H-18), 0.88 (s, H-19), 0.84 (s, H-30)], together with four *β*-glucopyranosyls [*δ*_H_ 4.39 (d, *J* = 7.7 Hz, H-1′), 4.61 (d, *J* = 7.8 Hz, H-1″), 4.37 (d, *J* = 7.7 Hz, H-1″′), 4.26 (d, *J* = 7.8 Hz, H-1″″)]. The ^13^C NMR (Table 1 and Table 2) and HSQC data showed resonances for 54 carbons, including two double bonds [*δ*_C_ 144.0 (C-5), 132.0 (C-25), 130.0 (C-24), 120.0 (C-6)], one oxymethylene [*δ*_C_ 76.4 (C-26)], one oxymethine [*δ*_C_ 88.0 (C-3)], four methines [*δ*_C_ 51.9 (C-17), 45.1 (C-10), 39.6 (C-8), 37.1 (C-20)], four quaternary carbons [*δ*_C_ 50.5 (C-9, 13), 48.1 (C-14), 42.5 (C-4)], and seven methyls [*δ*_C_ 28.8 (C-29), 28.7 (C-19), 26.1 (C-28), 18.9 (C-30), 16.0 (C-18), 14.1 (C-27)], together with four *β*-glucopyranosyls [*δ*_C_ 106.9 (C-1″″), 104.7 (C-1″′), 104.9 (C-1″), 101.4 (C-1′), 82.0, 78.2, 78.1, 78.0, 78.0, 78.0, 77.9, 77.7, 77.5, 76.7, 75.5, 75.1, 71.6, 71.5, 71.4, 71.2, 69.6, 62.7, 62.7, 62.6]. The above data suggested that compound **1** was a cucurbitane-type triterpene glycoside with four *β*-glucopyranosyls [8].

**Figure 2:**
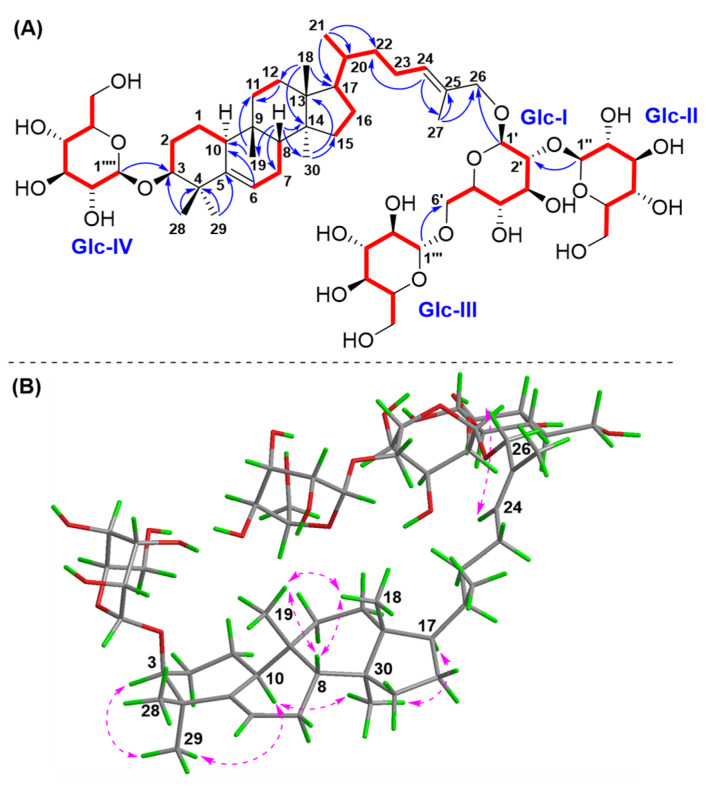
Key ^1^H-^1^H COSY (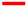), HMBC (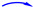), and NOESY (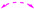) correlations of compound **1**. (**A**) ^1^H-^1^H COSY and HMBC; (**B**) NOESY.

The ^1^H-^1^H COSY correlations (Figure 2) of H-10/H_2_-1/H_2_-2/H-3, H-6/H_2_-7/H-8, H_2_-11/H_2_-12, H_2_-15/H_2_-16/H-17/H-20 (H_3_-21)/H_2_-22/H_2_-23/H-24, and the main HMBC (Figure 2) correlations from the methyls H_3_-18 to C-12, C-13, C-14, C-17; H_3_-19 to C-8, C-9, C-10, C-11; H_3_-28/29 to C-3, C-4, C-5; H_3_-30 to C-13, C-14, C-15; and H_3_-27 to C-24, C-25, C-26, allowed for the assignment of the cucurbitane triterpene nucleus structure of compound **1**. The observation of HMBC correlations from two olefinic protons H-6 to C-4, C-5, C-7, C-8, and C-10 and H-24 to C- 22, C-23, C-25, C-26, and Me-27 confirmed that two double bonds were present at Δ^6,7^ and Δ^24,25^, respectively. The obvious HMBC correlations from H_3_-28/H_3_-29 to C-3 (*δ*_C_ 88.0) and H-24/H_3_-27 to C-26 (*δ*_C_ 76.4) indicated a possible sugar moiety substitution or free hydroxyl group at C-3 and C-26, respectively.

All chemical shifts of each monosaccharide moiety (Table 2) were assigned based on the combination of HSQC, HMBC, and HSQC-TOCSY spectra [9,10]. In the TOCSY spectrum, the terminal protons of four *β*-glucopyranosyl units were obviously correlated with other corresponding protons of each one. The HMBC correlations from Glc II-H-1″ (*δ*_H_ 4.61) to Glc I-C-2′ (*δ*_C_ 82.0), Glc III-H-1″′ (*δ*_H_ 4.37) to Glc I-C-6′ (*δ*_C_ 69.6), and Glc I-H-1′ (*δ*_H_ 4.39) to C-26 (*δ*_C_ 76.4) established the saccharidic chain Glc III-(1→6)-[Glc II-(1→2)]-Glc I substituted at C-26 [11,12]. The obvious HMBC correlation from the terminal proton Glc IV-H-1″″ (*δ*_H_ 4.26) to C-3 (*δ*_C_ 88.0) allowed for the assignment of a *β*-glucopyranosyl Glc IV at C-3. Therefore, the planar structure of compound **1** was established.

**Figure 3:**
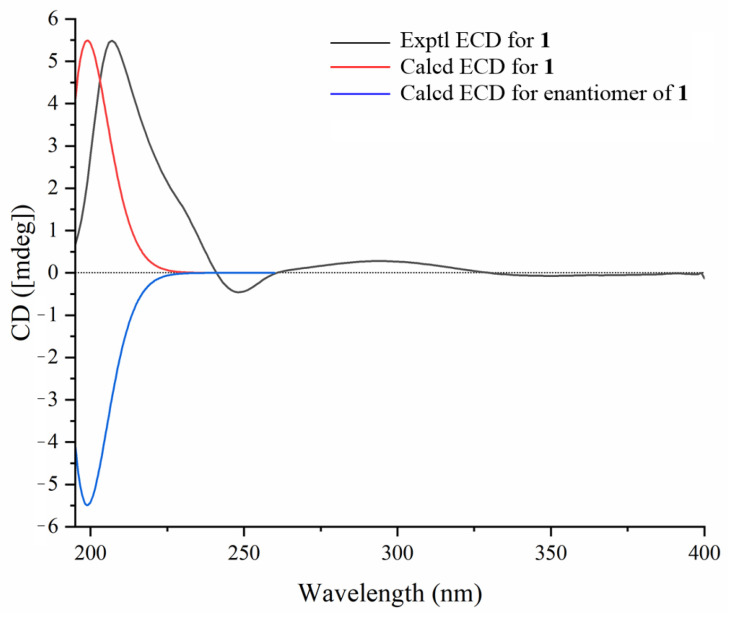
Experimental and calculated ECD spectra of compound **1**.

The relative configuration of compound **1** was determined through the analysis of characteristic proton–proton coupling constants and NOESY correlations (Figure 2). A broad singlet of H-3 at *δ*_H_ 3.40 (brs) suggested an equatorial configuration (*J*_3eq, 2ax_ ≌ *J*_3eq, 2eq_) of H-3, which combined with a biosynthetic analogy to related cucurbitane triterpenes supported *α*-orientation of H-3 [8,13]. The *α*-orientations of H-10, H-17, H_3_-29, and H_3_-30 were identified based on the key NOESY correlations of H-3/H-10 and H_3_-29 and H-10/H-17 and H_3_-30. The correlations of H-8 and H_3_-18/H_3_-19 suggested that these protons were *β*-orientations. The *E* configuration of Δ^24,25^ was confirmed based on the key NOESY correlations between H-24 and H_2_-26. Acid hydrolysis of compound **1** with 4 M HCl afforded **1a** and the glucoses, and the absolute configurations of D-glucoses were identified with HPLC-CAD analysis [27,28]. The ECD experiment (Figure 3) of compound **1a** suggested a positive Cotton effect at 207 nm (Δ*ε* 3.70), which was rather consistent with that of hemchinin F [10]. Furthermore, the calculated ECD curve of **1a** was also in good agreement with the experimental one (Figure 3), and thus the absolute configuration of compound **1** was confirmed as 3*S*, 8*R*, 9*R*, 10*S*, 13*R*, 14*S*, 17*R*, 20*R*. Accordingly, the structure of **1** was elucidated and named hemchinin G, as shown in Figure 1.

**Figure 4:**
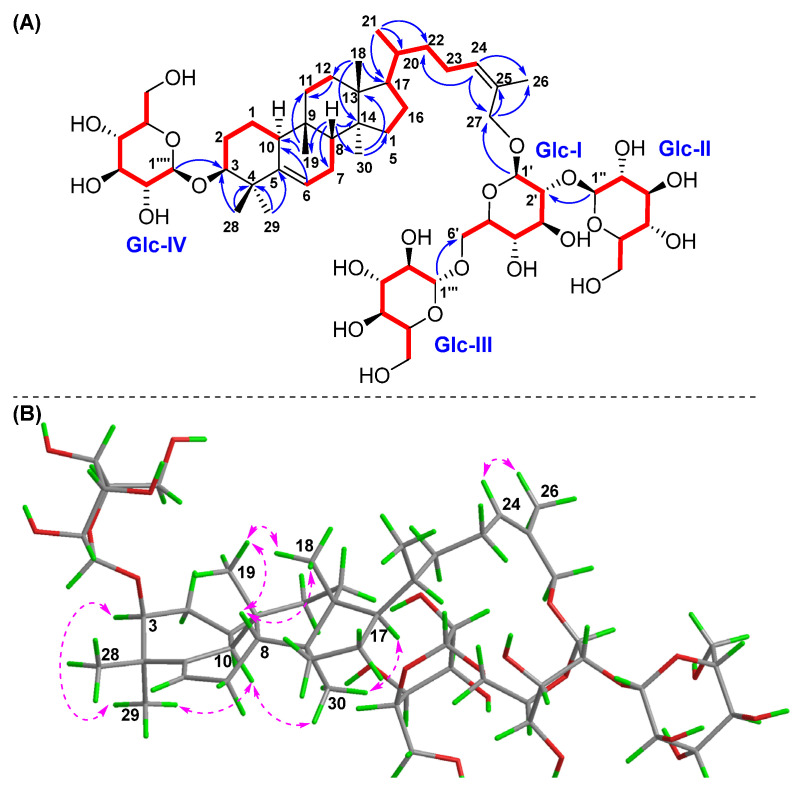
Key ^1^H-^1^H COSY (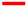), HMBC (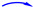), and NOESY (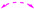) correlations of compound **2**. (**A**) ^1^H-^1^H COSY and HMBC; (**B**) NOESY.

**Figure 5:**
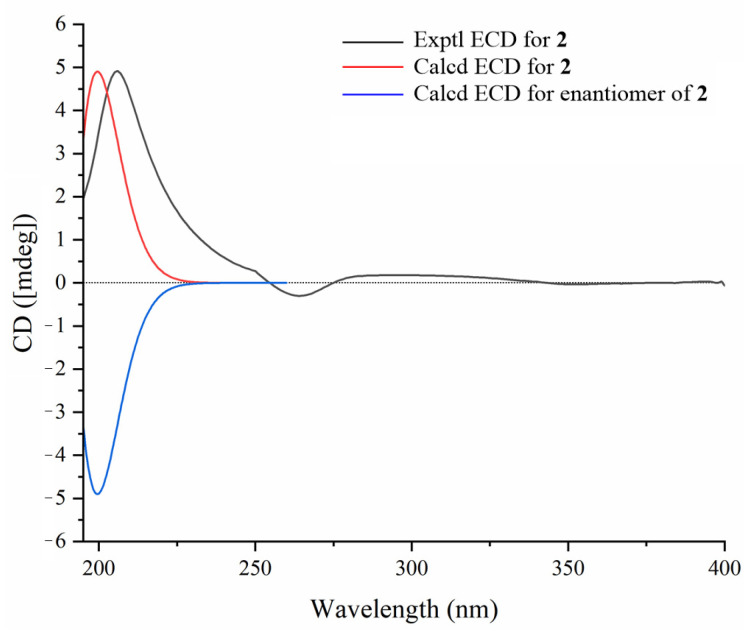
Experimental and calculated ECD spectra of compound **2**.

Compound **2** had the same molecular formula of C_54_H_90_O_22_ as **1** on the basis of a positive HR-ESI-MS ion at *m/z* 1113.5810 [M + Na]^+^ (calcd for C_54_H_90_O_22_Na, 1113.5816). The 1D and 2D NMR spectral data of compound **2** (Table 1 and Table 2) were also extremely similar to those of **1**, which indicated their similar structures. The difference was that the saccharidic chain Glc III-(1→6)-[Glc II-(1→2)]-Glc I in compound **2** was located at C-27 instead of C-26 in **1**, which was supported by the obvious chemical shift change of oxymethylene protons H_2_-27 [*δ*_H_ 4.26 (m, 2H)], and HMBC correlations (Figure 4) from H-1′ to C-27 and H_2_-27 to C-1′, as well as a key NOESY correlation (Figure 4) between H-24 and H_3_-26 [*δ*_H_ 1.79 (s)]. The *Z* configuration of Δ^24,25^ was accordingly confirmed by the NOESY cross-peak of H-24 and H_3_-26. The relative configuration of compound **2** was further determined based on characteristic proton–proton coupling constants and NOESY correlations as for those of **1**. Acid hydrolysis of compound **2** with 4 M HCl afforded **2a** and the D-glucoses, and the absolute configurations of D-glucoses were identified using HPLC-CAD [27,28]. Combined with ECD Cotton effects and calculation results of compound **2a** (Figure 5), the absolute configuration of **2** was elucidated as 3*S*, 8*R*, 9*R*, 10*S*, 13*R*, 14*S*, 17*R*, 20*R*. Therefore, the structure of **2** was confirmed and named hemchinin H, as shown in Figure 1.

**Figure 6:**
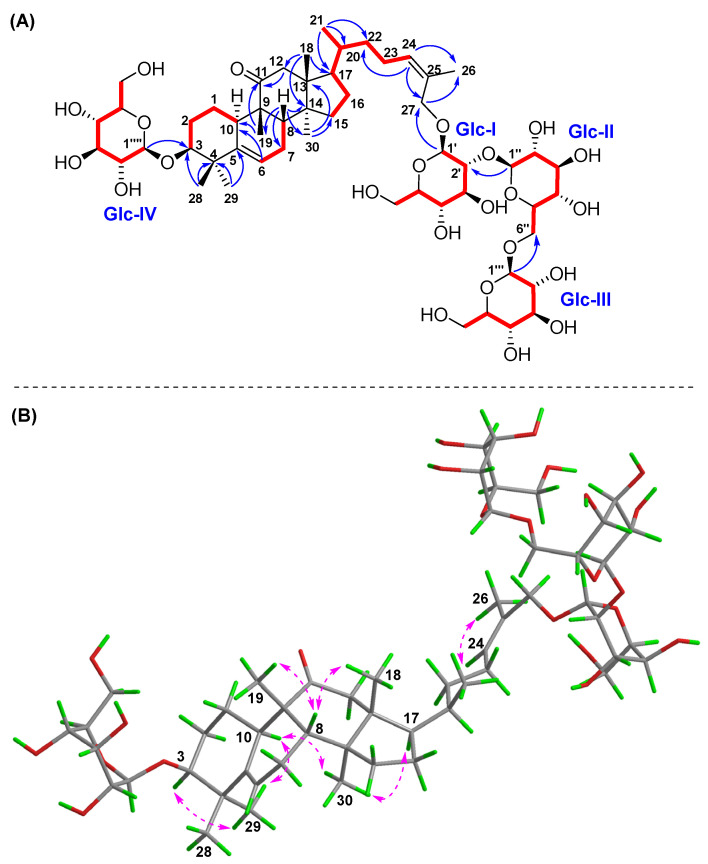
Key ^1^H-^1^H COSY (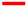), HMBC (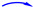), and NOESY (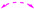) correlations of compound **3**. (**A**) ^1^H-^1^H COSY and HMBC; (**B**) NOESY.

**Figure 7:**
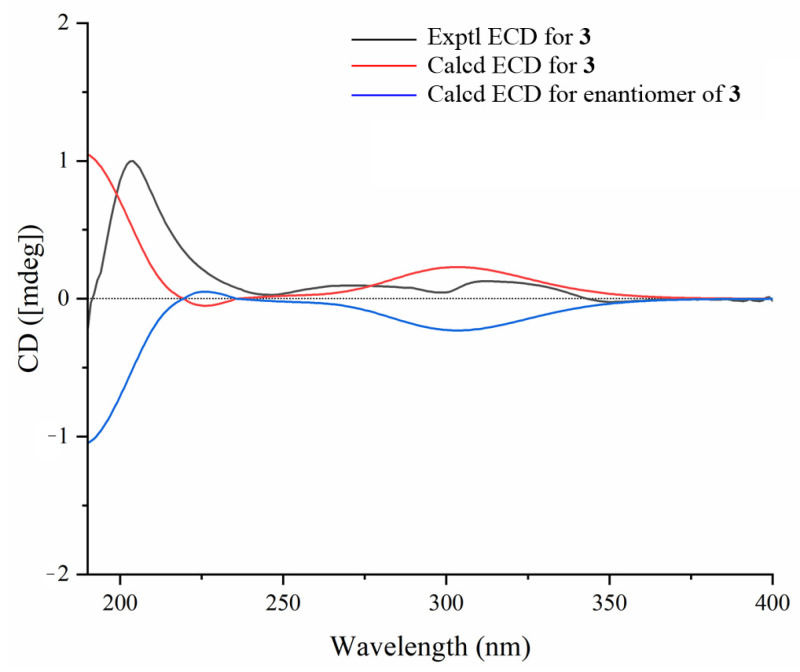
Experimental and calculated ECD spectra of compound **3**.

Compound **3** had a molecular formula of C_54_H_88_O_23_ based on the HR-ESI-MS spectrum at *m/z* 1127.5616 [M + Na]^+^ (calcd for C_54_H_88_O_23_Na, 1127.5608). According to the 1D and 2D NMR spectral data analyses (Table 1 and Table 2), the chemical structure of compound **3** was also a cucurbitane glycoside with four *β*-glucose groups, as with that of **1** and **2**. However, one main structural difference between compound **3** and that of **1** and **2** lay in the sequence of saccharidic chain Glc III-(1→6)-Glc II-(1→2)-Glc I, which was supported by the key HMBC correlations (Figure 6) from Glc III-H-1″′ (*δ*_H_ 4.47) to Glc II-C-6″, Glc II-H-1″ (*δ*_H_ 4.59) to Glc I-C-2′, and Glc I-H-1′ (*δ*_H_ 4.35) to C-26. And the saccharidic chain was located at C-27 as **2** due to the oxymethylene protons H_2_-27 [*δ*_H_ 4.28 (m, 2H)] and HMBC correlations from H-1′ to C-27 and H_2_-27 to C-1′, as well as an obvious NOESY correlation (Figure 6) between H-24 and H_3_-26 [*δ*_H_ 1.79 (s)]. Moreover, the final difference from compounds **1** and **2** was that a keto carbonyl group at C-11 (*δ*_C_ 218.0) in **3**, which was secured from the key HMBC cross-peaks from H-8/H-10/H_2_-12/H_3_-19 to C-11. The NOESY correlation between H-24 and H_3_-26 indicated the *Z* configuration of Δ^24,25^. The relative configuration of compound **3** was further determined based on characteristic coupling constants and NOESY correlations as those of **1**. The D-glucoses were identified through HPLC-CAD analysis after acid hydrolysis of compound **3** with 4 M HCl [27,28]. Combined with the ECD Cotton effects and calculation results (Figure 7) of the aglycone product **3a,** we confirmed its absolute structure as 3*S*, 8*S*, 9*R*, 10*R*, 13*R*, 14*S*, 17*R*, 20*R*. Therefore, the structure of compound **3** was determined as shown in Figure 1 and named hemchinin I.

Thirteen known cucurbitane triterpene glycosides with one to two glucose substitutions were isolated and identified, including hemslepenside A (**4**) [11], xuedanoside H (**5**) [12], scandenoside R1 (**6**) [13], scandenoside R2 (**7**) [14], carnosifloside II (**8**) [15], scandenoside R5 (**9**) [13], 3-*O*-*β*-D-glucopyranosyl-3β,27-dihydroxycucurbita-5,24(*Z*)-diene-11 -one-27-*O*-*β*-D-glucopyranoside (**10**) [15], jinfushanoside G (**11**) [16], hemslepenside I (**12**) [17], hemslepenside H (**13**) [17], 3*β*,11*α*,26-trihydroxy-cucurbita-5,24(E)-diene-3-*O*-*β*-D-glucopyranosyl-26-*O*-β-D-glucopyranosyl (1→6)-*α*-L-arabinoside (**14**) [18], hemsleyaoside M (**15**) [19], and carnosifloside I (**16**) [15], through comparison of the ^1^H and ^13^C NMR data with those reported.

### 2.2. Anti-Inflammatory Effects of Compounds ***1***–***3***

**Figure 8:**
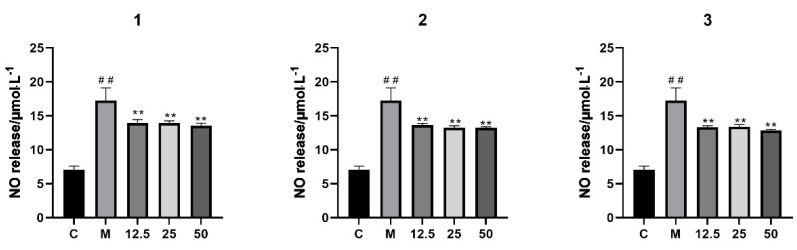
NO Inhibitory effects of compounds **1–3** in LPS-stimulated RAW264.7 cells ($\bar{x}$ ± s, *n* = 3). ## *p* < 0.001, compared with the control group; ** *p* < 0.001, compared with the model group.

Compounds **1**–**3** were evaluated for inhibitory activity against LPS-induced NO production in RAW 264.7 macrophages. No cytotoxicity was observed in compounds **1**–**3** at ≤100 μM toward RAW 264.7 cells (cell viability > 90%). Moreover, LPS-stimulated RAW 264.7 cells were used to investigate their anti-inflammatory properties at 12.5, 25, and 50 μM. According to the findings, compounds **1**–**3** all showed weak inhibitory activities against NO production (Figure 8).

### 2.3. Molecular Docking

**Figure 9:**
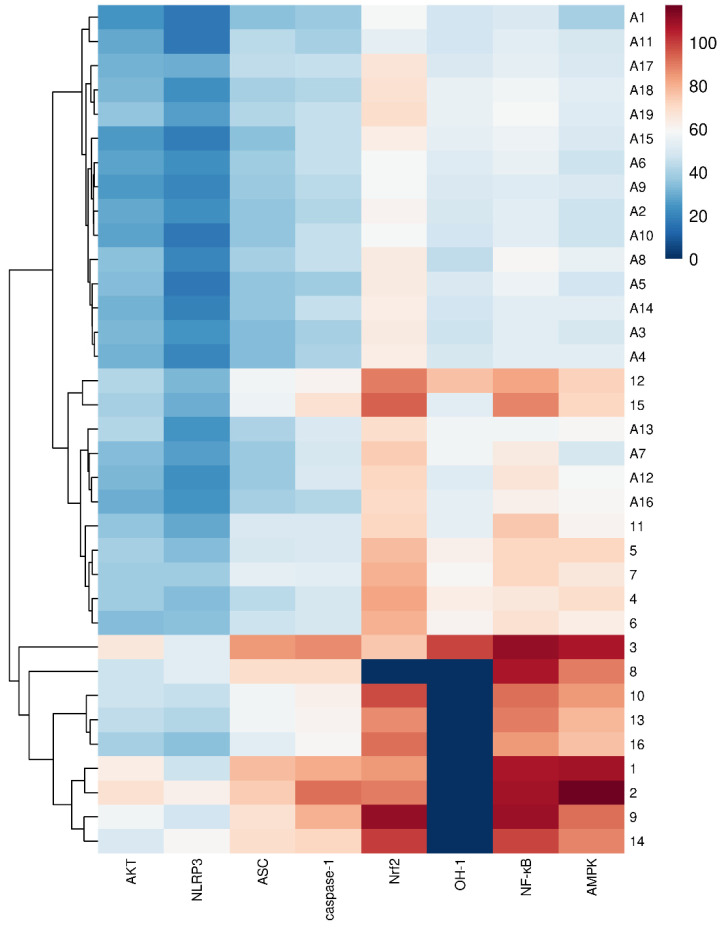
Heatmap of molecular docking.

**Figure 10:**
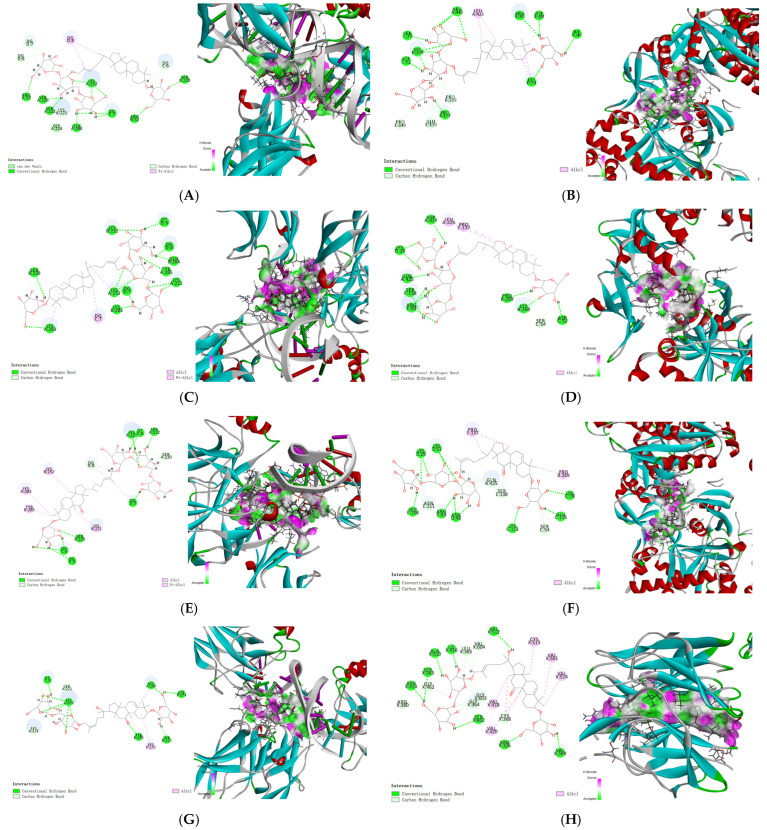
Molecular docking of NF-κB, AMPK and Nrf2 with typical compounds. (**A**,**C**,**E**,**G**) Molecular docking of NF-κB with compounds **1**–**3** and **9**; (**B**,**D**,**F**) molecular docking of AMPK with compounds **1**–**3**; and (**H**) molecular docking of Nrf2 with compound **9**.

All of the isolated cucurbitane glycosides **1**–**16**, together with nineteen reported cucurbitane triterpenes **A1**–**A19** [10] (Figure S31), including hemchinins A–F (**A1**–**A6**), cucurbitacin IIb (**A7**), cucurbitacin IIa (**A8**), scandenogenin A (**A9**), scandenogenin B (**A10**), jinfushanencins F (**A11**), 23,24-dihydrocucurbitacin B (**A12**), 23,24-dihydroisocucurbitacin B (**A13**), 22-deoxy-22-hydroxy-25-deacetoxy-cucurbitacin B (**A14**), 2*β*,16*α*,2*R*-trihydroxy-10*α*,17*α*-cucurbit-5,25-dien-3,11,22-trione (A**15**), dihydrocucurbitacin E (**A16**), 23,24-dihydrocu-curbitacin D (**A17**), isocucurbitacin R (**A18**), and jinfushanencin A (**A19**), were all docked to AKT (PDB ID: 2 × 18), NLRP3 (PDB ID: 8ERT), ASC (PDB ID: 6XKJ), caspase-1 (PDB ID: 6VIE), Nrf2 (PDB ID: 7K2F), OH-1 (PDB ID: 4WD4), NF-κB (PDB ID: 7CLI), and AMPK (PDB ID: 88IK). CDOCKER_INTERACTION_ENERGY ≤ −5.0 kJ/mol was used to produce a better binding ability for the compounds and proteins. The results are shown in Table S1, and Figure 9 and Figure 10.

The results showed that most of the compounds showed the highest binding energies with Nrf2, followed by NF-κB, and finally AMPK. Among them, compounds **9**, **10** and **14** have the highest binding energies with Nrf2, at −111.24, −97.30 and −100.02 kJ/mol, respectively; **1**–**3**, **8**–**9,** and **14** showed the highest binding energy with NF-κB, at −106.70, −108.86, −111.37, −106.74, −109.371, and −98.70 kJ/mol, respectively; and **1–3** have the highest binding energies with AMPK, at −108.54, −117.29 and −107.53 kJ/mol, respectively. What is more, it was easy to find that compounds **1**–**16** had higher binding energies with NF-κB, AMPK, Nrf2, caspase-1, ASC, AKT, and NLRP3. By observing the chemical structures, compounds **1**–**16** were all cucurbitane glycosides with one to four *β*-glucoses, especially when multiple glucoses were substituted at C-3 and C-26/27 simultaneously. Among them, compounds with the sugar substitution at C-26/27, especially two to three *β*-glucoses such as **1–3**, **8**–**9,** and **14**, clearly showed better binding capacities than those compounds without sugar substitution, such as **4**–**7** and **11**. Additionally, cucurbitane triterpenes possessing an acetyl group at C-25, such as compounds **A7**, **A12**, **A13**, and **A16**, exhibited better binding capacities than the other cucurbitane triterpenes, especially with NF-κB.

Among these compounds, cucurbitacin IIa (**A8**) and cucurbitacin IIb (**A7**) had the highest content in this species. Cucurbitane triterpenes and cucurbitane glycosides existed widely in the EtOAc and *n*-butanol fractions of this species, respectively. These results demonstrated that cucurbitane triterpenes and glycosides, as the main active components in *H. chinensis* tubers, showed high docking scores for affinities with proteins involved in the NF-κB, AMPK, and Nrf2 signaling pathways. Notably, cucurbitane triterpenes with sugar moieties substitution showed enhanced affinity for anti-inflammatory-related proteins, which indicated that cucurbitane glycosides are the important substance basis for the anti-inflammatory activity of *H. chinensis* tubers. Further activity screening and validation of cucurbitane glycosides, as well as mechanism of action research, will promote the discovery of novel anti-inflammatory lead compounds with low toxicity and good activity and lay the foundation for the rational development and utilization of *H. chinensis* tubers.

## 3. Materials and Methods

### 3.1. Materials and Reagents

Optical rotations were measured on an Anton Paar MCP 5100 polarimeter (Anton Paar, Ashland, VA, USA). UV spectra were recorded on a Shimadzu double-beam 210A spectrophotometer (Shimadzu, Tokyo, Japan). IR spectra were run on a Thermo Nicolet IS5 spectrometer (Thermo Fisher Scientific, Waltham, MA, USA). HR-ESI-MS data were obtained with a Thermo UPLC/Orbitrap Exploris 120 Spectrometer (Thermo Fisher Scientific, Waltham, MA, USA). One-dimensional and two-dimensional NMR spectra were obtained on a Bruker Avance III 500-NMR instrument (Bruker, Billerica, MA, USA). The ECD spectra were recorded with an Applied Photophysics Chirascan V100 spectrometer (Applied Photophysics, Leatherhead, UK). Silica gel (100–200 and 200–300 mesh, Qingdao Marine Chemical Inc., Qingdao, China), ODS-C18 (50 μM, YMC Co., Ltd., Kyoto, Japan), and semi-prep HPLC (QBH LC-52, Beijing Qingbohua Technology Co., Ltd., Beijing, China) with a YMC (250 mm × 10 mm, I.D. 5 μM) column were used to isolate the constituents.

### 3.2. Plant Material

The tubers of *Hemsleya chinensis* were purchased from Guizhou province of China in November 2022 and identified by Professor Liping Dai of Henan University of Chinese Medicine. The voucher specimen (No. 2020-1101) was deposited in the Henan Collaborative Innovation Center for Research and Development on the Whole Industry Chain of Yu-Yao.

### 3.3. Extraction and Isolation

The dried tubers (20.0 kg) were cut into small pieces and refluxed with 75% ethanol (2 × 105 L × 2 h). After removing the solvent, 2.3 kg of residue was respectively extracted with EtOAc and *n*-butanol to give two fractions (213.7 g and 381.8 g, respectively).

The remaining extracts from the EtOAc fraction were combined and subjected to an ODS column with a methanol–water (30% → 90%) gradient elution, resulting in four fractions, Fr. H-1~4. Fr. H-4 (5.1 g) was further separated through ODS column chromatography with methanol–water (10% → 30%) to obtain four subfractions, Fr. H-4-A~D. Compounds **6** (4.7 mg, *t_R_* = 132.5 min, 3 mL/min) and **7** (145.7 mg, *t_R_* = 110.5 min) were purified from Fr. H-4-B (333.2 mg) through semi-preparative HPLC (12% ACN-H_2_O). Compounds **14** (10.3 mg, *t_R_* = 68.1 min) and **9** (23.4 mg, *t_R_* = 99.5 min) were purified from Fr. H-4-D (1.0 g) via semi-preparative HPLC (20% ACN-H_2_O).

The *n*-butanol fraction was chromatographed using silica gel (100–200 mesh), eluted with a gradient of a DCM-MeOH system (1:0 → 0:1) to give 6 fractions. Fr. 2 (2.0 g) was separated using an ODS column using an acetonitrile–water (15% → 35%) gradient elution, yielding four fractions, Fr. 2-A~D. Compounds **4** (7.6 mg, *t_R_* = 153.5 min) and **5** (7.5 mg, *t_R_* = 189.4 min) were respectively obtained from Fr. 2-B (254.0 mg) and Fr. 2-D (138.2 mg) through semi-preparative HPLC purification (35% and 45% MeOH-H_2_O, respectively). Fr. 4 (6.5 g) was separated with an ODS column using an acetonitrile–water (23% → 45%) gradient elution, yielding four fractions, Fr. 4-A~D. Compound **13** (5.4 mg, *t_R_* = 155.7 min) was obtained from Fr. 4-A (320.5 mg) via semi-preparative HPLC purification (18% ACN-H_2_O). Compounds **10** (5.4 mg, *t_R_* = 60.3 min), **12** (5.0 mg, *t_R_* = 65.2 min), **15** (7.0 mg, *t_R_* = 93.5 min), and **16** (3.0 mg, *t_R_* = 135.5 min) were isolated from Fr. 4-B (1.2 g) through semi-preparative HPLC (22% ACN-H_2_O). Fr. 5 (9.9 g) was separated by ODS column using an acetonitrile–water (30% → 60%) gradient elution, yielding four fractions, Fr. 4-A~D. Compounds **11** (3.4 mg, *t_R_* = 88.5 min) and **8** (5.4 mg, *t_R_* = 125.5 min) were obtained from Fr. 4-C (320.5 mg) via semi-preparative HPLC purification (40% ACN-H_2_O). Fr.6 (103.0 g) was separated using an MCI column, eluted with a gradient of methanol–water (10% → 80%), to obtain Fr. 6-A~H. Fr. 6-C (8.2 g) was separated with an ODS column, eluted with a methanol–water (30% → 55%) system gradient to obtain Fr. 6-C-1~8. Compound **1** (4.2 mg, *t_R_* = 100.5 min) was purified from Fr. 6-C-8 (80.2 mg) through semi-preparative HPLC (40% MeOH-H_2_O). Fr.6-D (10.3 g) was separated using an ODS CC with methanol–water (50% → 70%), giving Fr. 6-D-1~8. Fr. 6-D-6 (2.5 g) was separated with an ODS column with methanol–water (60% → 70%) to obtain Fr. 6-D-6-1~4. Fr. 6-D-6-2 (200 mg) was purified through semi-preparative HPLC (32% MeOH-H_2_O) to give compounds **2** (10.1 mg, *t_R_* = 110.5 min, 3 mL/min) and **3** (17.3 mg, *t*_R_ = 210.5 min).

### 3.4. Spectral Data

Hemchinin G (**1**): yellow amorphous solid; [*α*${]}_{D}^{20}$ 13.000 (*c* 0.1, MeOH); IR (MeOH) *ν*_max_: 3385, 2918 and 1038 cm^−1^; UV (MeOH) *λ*_max_ (log ε): 204 (4.68) nm; ECD (MeOH) *λ*_max_ (Δε): 207 (3.70), 248 (−0.31) nm; ^1^H (500 MHz) and ^13^C (125 MHz) NMR data, see Table 1 and Table 2; HR-ESI-MS at *m/z* 1113.5815 [M + Na]^+^ (Calcd for C_54_H_90_O_22_, 1113.5816).

Hemchinin H (**2**): yellow amorphous solid; [*α*${]}_{D}^{20}$ 26.668 (*c* 0.1, MeOH); IR (MeOH) *ν*_max_*:* 3341, 2934, 1380, 1084 and 1024 cm^−1^; UV (MeOH) *λ*_max_ (log ε): 204 (4.68) nm; ECD (MeOH) *λ*_max_ (Δε): 206 (3.31), 264 (−0.20) nm; ^1^H (500 MHz) and ^13^C (125 MHz) NMR data, see Table 1 and Table 2; HR-ESI-MS at *m/z* 1113.5810 [M + Na]^+^ (Calcd for C_54_H_90_O_22_, 1113.5816).

Hemchinin I (**3**): white amorphous solid; [*α*${]}_{D}^{20}$ 3.000 (*c* 0.1, MeOH); IR (MeOH) *ν*_max_: 3363, 2832, 1662, 1453 and 1026 cm^−1^; UV (MeOH) *λ*_max_ (log ε): 203 (4.44) nm; ECD (MeOH) *λ*_max_ (Δε): 204 (0.68), 246 (0.02), 299 (0.03) nm; ^1^H (500 MHz) and ^13^C (125 MHz) NMR data, see Table 1 and Table 2; HR-ESI-MS at *m/z* 1127.5616 [M + Na]^+^ (Calcd for C_54_H_88_O_23_, 1127.5608).

### 3.5. Acid Hydrolysis for Compounds ***1***–***3***

A part of compounds **1**–**3** (2.0 mg) was hydrolyzed with 3 mL of 2 M HCl at 80 °C for 2.5 h. After extraction with ethyl acetate (3 × 1 mL), the aqueous layer was evaporated to furnish the monosaccharide residue. After purification of these sugars in the same solvent, D-glucoses for compounds **1**–**3** were identified using the HPLC-CAD method by comparing retention times with those of chemical reference standards D-glucose and L-glucose [15].

### 3.6. ECD Calculations of Compounds ***1a***–***3a***

The 3D structures were first established on the basis of the NOESY spectra. Their energy-minimized conformers were generated via the Molecular Mechanics field in Spartan 14. The conformers with a Boltzmann population of over 10% were chosen for ECD calculations. The predominant conformers of compounds **1a**–**3a** were subjected to ECD calculation with the TDDFT method at the rb3lyp/6-31 g(d,p) level using Gaussian 16. Their ECD curves were produced via OriginPro 8 with UV correction.

### 3.7. Anti-Inflammatory Bioassays

#### 3.7.1. Cell Culture and Cell Viability Assay

The RAW 264.7 cells (Procell Life Science & Technology Co. Ltd., Wuhan, China) were cultured in Dulbecco’s modified Eagle’s medium (DMEM) with 10% fetal bovine serum (FBS), 100 U/mL penicillin, and 100 U/mL streptomycin at 37 °C in a humidified atmosphere with 5% CO_2_. Cell viability was evaluated using MTT. The assay was performed by culturing the cells (1 × 10^5^ cells/mL) in 96-well plates for 12 h, followed by treatment with the tested compounds **1**–**3** in different concentrations (12.5, 25, and 50 μM) for 24 h. After adding 20 μL of MTT (5 mg/mL) to each well, the samples were incubated for 4 h. Removing the liquid, 150 μL DMSO was added to each well and the plate was shaken on an oscillator for 10 min to completely dissolve the formazan crystals. And the absorbance at 490 nm was measured by using a microplate reader.

#### 3.7.2. LPS-Induced NO Content in RAW 264.7 Cells

NO production was determined by measuring the nitrite concentration in the culture supernatant using Griess reagent. The cells (2 × 10^6^ per well) were seeded in 12-well plates for 12 h and then treated with compounds **1**–**3** at 12.5, 25, and 50 μM for 1 h. Dexamethasone (Sigma, St. Louis, MO, USA) was used as the positive control. Net, 1 μg/mL LPS was added and cultured for 24 h. In addition, 50 μL of the supernatant from incubates was mixed with an equal volume of Griess reagent (1% sulfanilamide in 5% phosphoric acid; 0.1% naphthyl ethylenediamine dihydrochloride in distilled water) at 37 °C. After 10 min, the absorbance was measured at 540 nm. NO inhibition rates were calculated.

### 3.8. Molecular Docking

Discovery Studio 2020 software was used to predict the docking of small-molecule compounds with key proteins. The 3D structure of the compound constructed using ChemBioOffice 2014 was saved in *mol2 format, and its energy was minimized. The 3D structure of the target protein was downloaded from the PDB data (https://www.rcsb.org/), and Discovery Studio 2020 software was used to perform operations such as water removal and hydrogenation on the protein and generate an effective single 3D conformation by minimizing the energy.

## 4. Conclusions

In this study, sixteen cucurbitane glycosides with one to four *β*-glucoses at C-3 and/or C-26/27, including three previously undescribed ones that were characterized by one saccharidic chain Glc III-(1→6)-[Glc II-(1→2)]-Glc I or Glc III-(1→6)-Glc II-(1→2)-Glc I substituted at C-26/27, were isolated from 70% ethanol extraction of *H. chinensis* tubers. The discovery of these three cucurbitane glycosides enriched the diversity of cucurbitane-type triterpenes in *H. chinensis*. In vitro anti-inflammatory assays revealed that compounds **1**–**3** showed certain NO inhibitions on LPS-induced RAW 264.7 cells. In addition, the isolated cucurbitane glycosides **1**–**16** and previously reported cucurbitane triterpenes **A1**–**A19** were docked to AKT, NLRP3, ASC, caspase-1, Nrf2, OH-1, NF-κB, and AMPK, and the results showed that cucurbitane triterpenes and glycosides, as the main active components, in *H. chinensis* tubers showed high docking scores of affinities with the proteins in the NF-κB, AMPK, and Nrf2 signaling pathways, which fully aligned with the previous finding that the ethanolic extract of *H. chinensis* tubers can exert protective effects on HCl/EtOH-induced acute gastric ulcers in rats, by inhibiting inflammatory mediators via the p38 MAPK/NF-κB pathway. Notably, cucurbitane triterpenes with sugar moieties substitution showed enhanced affinity to anti-inflammatory-related proteins. Structure–activity relationship analysis indicated that the active functional groups were related to the multiple glucose units at C-3 and/or C-26/27 positions, along with an acetyl group at C-25. A further in-depth investigation into the activity validation and mechanisms of action of cucurbitane glycosides will facilitate the discovery of novel anti-inflammatory lead compounds, thereby providing a foundation for the rational development and utilization of *H. chinensis* tubers.

## Figures and Tables

**Table 1:** The ^1^H (500 MHz) and ^13^C (125 MHz) NMR data for the aglycones of compounds **1–3** in CD_3_OD (*δ* in ppm and *J* in Hz).

NO.	1		2		3	
	*δ*_H_	*δ*_C_	*δ*_H_	*δ*_C_	*δ*_H_	*δ*_C_
1	1.49 (m) 1.54 (m)	23.2	1.50 (m) 1.54(m)	23.3	1.93 (m) 2.14 (m)	25.5
2	1.82 (m) 2.08 (m)	29.4	1.91 (m) 2.07 (m)	29.4	2.00 (m) 2.37 (m)	24.9
3	3.40 (brs)	88.0	3.42 (brs)	88.0	3.40 (brs)	88.0
4	-	42.5	-	42.6	-	42.9
5	-	144.0	-	141.0	-	141.2
6	5.51 (m)	120.0	5.52 (d, 5.7)	119.0	5.61 (d, 5.6)	119.0
7	1.77 (m) 2.36 (m)	25.3	1.80 (m) 2.37 (m)	25.4	1.33 (m) 2.00 (m)	29.2
8	2.29 (m)	39.6	2.28 (m)	39.6	1.93 (m)	45.4
9	-	50.5		49.6	-	50.0
10	1.75 (m)	45.1	1.75 (m)	45.2	2.40 (m)	36.9
11	1.52 (m) 1.71 (m)	31.6	1.44 (m) 1.69 (m)	33.4	-	217.0
12	1.45 (m) 1.69 (m)	33.4	1.15 (m) 1.25 (m)	35.5	2.37 (d, 14.3) 3.05 (d, 14.3)	50.1
13	-	50.5	-	50.5	-	50.5
14	-	48.1	-	47.5	--	48.1
15	1.15 (m) 1.25 (m)	35.8	1.15 (m) 1.23 (m)	36.0	1.30 (m) 1.37 (m)	35.4
16	1.29 (m) 1.94 (m)	29.0	1.52 (m) 1.71 (m)	31.6	1.06 (m) 1.45 (m)	37.6
17	1.53 (m)	51.9	1.49 (m)	52.1	1.75 (m)	50.5
18	0.89 (s)	16.0	0.88 (s)	16.1	0.73 (s)	17.2
19	0.88 (s)	28.7	0.87 (s)	28.4	1.05 (s)	20.0
20	1.34 (m)	37.1	1.45 (m)	37.3	1.43 (m)	37.2
21	0.93 (d, 6.0)	19.4	0.93 (d, 6.0)	19.5	0.93 (d, 6.4)	18.9
22	1.48 (m) 1.51 (m)	37.2	1.04 (m) 1.46 (m)	37.8	1.44 (m) 2.40 (m)	37.1
23	1.95 (m) 2.10 (m)	25.5	1.96 (m) 2.10 (m)	25.8	1.97 (m) 2.14 (m)	26.0
24	5.48 (m)	130.0	5.34 (t, 5.2)	132.1	5.35 (m)	132.7
25	-	132.0	-	130.0	-	129.0
26	4.01 (d, 11.7) 4.21 (d, 11.7)	76.4	1.79 (s)	22.1	1.79 (s)	22.0
27	1.67 (s)	14.1	4.26 (m)	68.5	4.27 (m)	68.1
28	1.19 (s)	26.1	1.19 (s)	26.1	1.22 (s)	26.1
29	1.02 (s)	28.8	1.02 (s)	28.8	1.06 (s)	28.9
30	0.84 (s)	18.9	0.84 (s)	18.5	1.08 (s)	19.1

**Table 2:** The ^1^H (500 MHz) and ^13^C (125 MHz) NMR data for the sugar moieties of compounds **1–3** in CD_3_OD (*δ* in ppm and *J* in Hz).

NO.	1		2		3	
	*δ*_H_	*δ*_C_	*δ*_H_	*δ*_C_	*δ*_H_	*δ*_C_
Glc-I						
1	4.39 (d, 7.7)	101.4	4.28 (d, 7.8)	101.6	4.35 (d, 7.5)	101.2
2	3.48 (m)	82.0	3.17 (m)	82.0	3.57 (m)	82.0
3	3.40 (m)	76.7	3.20 (m)	76.7	3.37 (m)	71.4
4	3.25 (m)	71.6	3.23 (dt, 8.6, 3.0)	71.4	3.24 (m)	75.5
5	3.54 (t, 6.2)	77.9	3.24 (dt, 8.6, 3.0)	78.1	3.21 (m)	75.3
6	3.77 (m) 4.13 (d, 11.3)	69.6	3.68 (dt, 11.6, 5.5) 3.85 (m)	69.7	3.64 (m) 3.82 (d, 2.3)	62.5
Glc-II						
1	4.61 (d, 7.8)	104.9	4.61 (d, 7.5)	104.7	4.59 (d, 7.9)	104.6
2	3.21 (m)	75.5	3.23 (m)	75.9	3.26 (m)	77.9
3	3.27 (m)	78.0	3.52 (m)	78.0	3.19 (m)	77.8
4	3.42 (m)	71.2	3.41 (q, 3.6)	71.6	3.34 (m)	78.3
5	3.36 (m)	78.0	3.37 (m)	77.8	3.45 (m)	77.5
6	3.62 (m) 3.75 (m)	62.7	3.67 (dt, 11.6, 5.4) 3.82 (m)	62.8	3.77 (d, 5.8) 4.09 (d, 2.1)	69.6
Glc-III						
1	4.37 (d, 7.7)	104.7	4.34 (d, 7.9)	104.9	4.47 (d, 7.8)	104.6
2	3.22 (m)	78.0	3.21 (d, 1.4)	75.2	3.18 (m)	75.1
3	3.54 (t,6.2)	77.7	3.35 (dd, 8.6, 1.9)	77.7	3.23 (dd, 7.1, 1.8)	78.0
4	3.28 (m)	71.5	3.27 (dt, 8.6, 3.0)	71.6	3.28 (s)	71.5
5	3.37 (m)	77.5	3.41 (m)	76.9	3.42 (d, 3.8)	77.5
6	3.68 (m) 3.85 (m)	62.7	3.65 (dt, 11.6, 5.4) 3.80 (m)	62.8	3.69 (d, 5.5) 3.85 (s)	62.7
Glc-IV						
1	4.26 (d, 7.8)	106.9	4.28 (d, 7.8)	106.7	4.26 (d, 7.3)	106.4
2	3.17 (d, 8.3)	75.1	3.17 (m)	75.1	3.16 (m)	75.6
3	3.31 (m)	78.1	3.20 (m)	75.6	3.20 (m)	78.2
4	3.29 (d)	71.4	3.23 (dt, 8.6, 3.0)	71.7	3.28 (m)	71.6
5	3.34 (m)	78.2	3.24 (dt, 8.6, 3.0)	77.6	3.23 (m)	77.8
6	3.69 (m) 3.89 (d, 2.0)	62.6	3.68 (dt, 11.6, 5.5) 3.85 (m)	62.9	3.66 (m) 3.89 (s)	62.7

## Data Availability

Data are contained within the article or Supplementary Materials.

## Supplementary Materials

The following supporting information can be downloaded at https://www.mdpi.com/article/10.3390/molecules30112349/s1. Full spectroscopic spectra (MS, IR, UV, 1D, and 2D NMR) for compounds **1**–**3**, see Figures S1–S33; The structures of compounds **A1**–**A19**, see Figure S34; Binding affinity of different compounds to inflammatory pathway-related target proteins, see Table S1; The optimized lowest energy 3D conformers and energy analysis of compounds **1a**–**3a**, see Table S2.

## References

[1] China Flora Editorial Board (Chinese Academy of Sciences) (1986). Flora of China.

[2] Dong J.Y., Yue L., Shao W.W., Xu J.Z. (2012). Chemical components in *Hemsleya chensnsis* (III). China J. Chin. Mater. Med..

[3] Li X.S., Wang Q.L., Xu Z.P., Liu M.S., Liang X.Y., Zheng Jia C., Deng H.Y., Liu L.i., Huang Y.M., Yang M.X. (2024). Structurally diverse cucurbitane-type triterpenoids from the tubers of *Hemsleya chinensis* with cytotoxic activity. Phytochemistry.

[4] Guizhou Medical Products Administration (2019). Standards for Traditional Chinese Medicine Ethnic Medicinal Materials in Guizhou Province.

[5] Hubei Medical Products Administration (2018). Quality Standards for Traditional Chinese Medicine in Hubei Province.

[6] Zhang Y., Guo H., Lian F.H., Xiang X., Li Q.X., Liu X.Q., Wang Z.M., Dai L.P., Xu E.P. (2023). Protective effect of ethanolic extract of *Hemsleya chinensis* on HCl/ethanol-induced acute gastric ulcer in rats based on p38MAPK/NF-kB signaling pathway. Chin. J. Exp. Tradit. Med. Formulae.

[7] Haq F.U., Ali A., Khan M.N., Shah S.M.Z., Kandel R.C., Aziz N., Adhikari A., Choudhary M., Atta-Ur-Rahman, El-Seedi H.R. (2019). Metabolite profiling and quantitation of cucurbitacins in cucurbitaceae plants by liquid chromatography coupled to tandem mass spectrometry. Sci. Rep..

[8] Chen J.C., Chiu H.M., Nie L.R., Geoffrey A.C., Samuel X.Q. (2005). Cucurbitacins and cucurbitane glycosides: Structures and biological activities. Nat. Prod. Rep..

[9] Yu K., Liu A.G., Liu B., Yao Q.Q. (2019). Progress on cucurbitacin IIa and its derivatives. Food Drug..

[10] Lian F.H., Chi J., Meng Q.L., Li Q.X., Chen A.Y., Wang Z.M., Dai L.P. (2023). Cucurbitane triterpenes from *Hemsleya chinensis* tubers and their anti-inflammatory activities. Fitoterapia.

[11] Xu X., Bai H., Zhou L., Deng Z., Zhong H., Wu Z., Yao Q. (2014). Three new cucurbitane triterpenoids from *Hemsleya penxianensis* and their cytotoxic activities. Bioorg. Med. Chem. Lett..

[12] Chen D.L., Xu X.D., Li R.T., Wang B.W., Yu M., Liu Y.Y., Ma G.X. (2019). Five new cucurbitane-type triterpenoid glycosides from the rhizomes of *Hemsleya penxianensis* with cytotoxic activities. Molecules.

[13] Kasai R., Matsumoto K., Nie R.L., Zhou J., Tanaka O. (1988). Glycosides from Chinese medicinal plant, *Hemsleya panacis-scandens*, and structure-taste relationship to cucurbitane glycosides. Chem. Pharm. Bull..

[14] Wei H., Liu Y.H., Tian Y.G., Xiang F.F. (2018). Cucurbitane-type triterpenoids from the tubers of *Hemsleya dolichocarpa*. Acta Pharm. Sin..

[15] Kasai R., Matsumoto K., Nie R.L., Morita T., Awazu A., Zhou J., Tanaka O. (1987). Sweet and bitter cucurbitane glycosides from *hemsleya carnosiflora*. Phytochemistry.

[16] Chen J.C., Zhou L., Wang Y.H., Tian R.R., Yan Y.X., Nian Y., Sun Y., Zheng Y.T., Qiu M.H. (2012). Cucurbitane triterpenoids from *Hemsleya penxianensis*. Nat. Prod. Bioprospect..

[17] Li Y., Wang W.X., Zheng Z.F., Mu Y.L., Liu Y.J., Wang H.Y., Li L., Yao Q.Q. (2018). Eight new cucurbitane triterpenoids from “Xue Dan”, the roots of *Hemsleya pengxianensis*. J. Asian Nat. Prod. Res..

[18] Wang W.X., Mu Y.L., Li Y., Zheng Z.F., Chu H.P., Zhou L., Yao Q.Q. (2018). Three new cucurbitane-type triterpenoid saponins from tubers of *Hemsleya pengxianensis* var. jinfushanensis. Chin. Trad. Herb. Drugs.

[19] Li Z., Chen M., Chen F., Li W., Huang G., Xu X., Wang S., Ma G., Cui P. (2022). Cucurbitane triterpenoid entities derived from *Hemsleya penxianensis* triggered glioma cell apoptosis via ER stress and MAPK signalling cross-talk. Bioorg. Chem..

[20] Liu T., Zhang L., Joo D., Sun S.C. (2017). NF-κB signaling in inflammation. Signal Transduct. Target. Ther..

[21] Gałgańska H., Jarmuszkiewicz W., Gałgański Ł. (2023). Carbon dioxide and MAPK signalling: Towards therapy for inflammation. Cell Commun. Signal..

[22] Manning B.D., Toker A. (2017). AKT/PKB signaling: Navigating the network. Cell.

[23] Swanson K.V., Deng M., Ting J.P.Y. (2019). The NLRP3 inflammasome: Molecular activation and regulation to therapeutics. Nat. Rev. Immunol..

[24] Li B.Y., Wang Y.X., Jiang X.L., Du H.W., Shi Y., Xiu M.H., Liu Y.Q., He J.Z. (2023). Natural products targeting Nrf2/ARE signaling pathway in the treatment of inflammatory bowel disease. Biomed. Pharmacother..

[25] Echizen K., Hirose O., Maeda Y., Oshima M. (2016). Inflammation in gastric cancer: Interplay of the COX-2/prostaglandin E2 and Toll-like receptor/MyD88 pathways. Cancer Sci..

[26] Zhang Y.E. (2017). Non-Smad pathways in TGF-β signaling. Cell Res..

[27] Meng Q.L., Chi J., Li Y.X., Zhang W.J., Zhang L.X., Wang Z.M., Dai L.P. (2024). Diarylheptanoid glycosides from *Dioscorea nipponica* rhizomes. Fitoterapia.

[28] Zhang Y., Yu H.Y., Chao L.P., Qu L., Ruan J.Y., Liu Y.X., Dong Y.Z., Han L.F., Wang T. (2016). Anti-inflammatory steroids from the rhizomes of *Dioscorea septemloba* Thunb. Steroids.

