# Supplementary material for: Cucurbitane Glycosides and Their Potential Anti-Inflammatory Activities from Hemsleya chinensis Tubers
Source: Molecules. 2025 May 28;30(11):2349. doi: 10.3390/molecules30112349 (PMC12156364; doi:10.3390/molecules30112349)
Supplement: Supplementary file 1 [file molecules-30-02349-s001.zip › molecules-3565514-supplementary.pdf]

Supplementary Materials

Cucurbitane Glycosides and Their Potential Anti-Inflammatory Activities from *Hemsleya chinensis* Tubers

Jun Chi [1,2], Miaomiao Li [1,2], Feihe Lian [1,2], Yixiao Li [1,2] and Liping Dai [1,2,*]

[1] Henan Collaborative Innovation Center for Research and Development on the Whole Industry Chain of Yu-Yao, Henan University of Chinese Medicine, Zhengzhou 450046, China; chijun16@126.com (J.C.); 1918290380@163.com (M.L.); 15738349001@163.com (F.L.); liyixiao0709@163.com (Y.L.)

[2] Engineering Technology Research Center for Comprehensive Development and Utilization of Authentic Medicinal Materials in Henan Province, Henan University of Chinese Medicine, Zhengzhou 450046, China

* Correspondence: liping_dai@hactcm.edu.cn; Tel.: +86-18703651652

List of Contents

Figure S1. HR-ESI-MS spectrum of compound **1** in MeOH………………….……………………4

Figure S2. IR spectrum of compound **1** in MeOH………………………………….……………5

Figure S3. UV spectrum of compound **1** in MeOH……………………………………………….6

Figure S4. ^{1}H NMR (500 MHz) spectrum of compound **1** in MeOD………………………………7

Figure S5. ^{13}C NMR (125 MHz) spectrum of compound **1** in MeOD……………………...……….8

Figure S6. gCOSY spectrum of compound **1** in MeOD…………………………...………….…...9

Figure S7. HSQC spectrum of compound **1** in MeOD…..……………………………………..10

Figure S8. HMBC spectrum of compound **1** in MeOD……………………….……...………..11

Figure S9. The enlarged HMBC spectrum of compound 1 in 0.7~1.9 ppm in MeOD...………....12

Figure S10. NOESY spectrum of compound **1** in MeOD…………………………...……….....13

Figure S11. TOCSY spectrum of compound **1** in MeOD…………………………..…...………..14

Figure S12. HR-ESI-MS spectrum of compound **2** in MeOH…………………….…..…...………..15

Figure S13. IR spectrum of compound **2** in MeOH……………………………..…...………..16

Figure S14. UV spectrum of compound **2** in MeOH………………………………………………..17

Figure S15. ^{1}H NMR (500 MHz) spectrum of compound **2** in MeOD……………………….………18

Figure S16. ^{13}C NMR (125 MHz) spectrum of compound **2** in MeOD…………………....………19

Figure S17. gCOSY spectrum of compound **2** in MeOD……………………….....…………….20

Figure S18. HSQC spectrum of compound **2** in MeOD……………………………...………..21

Figure S19. HMBC spectrum of compound **2** in MeOD…………………………..…...………..22

Figure S20. The enlarged HMBC spectrum of compound **2** in 0.6~2.0 ppm in MeOD...………..23

Figure S21. NOESY spectrum of compound **2** in MeOD…………………………….....………..24

Figure S22. TOCSY spectrum of compound **2** in MeOD…………………………….....………..25

Figure S23. HR-ESI-MS spectrum of compound **3** in MeOH…………….…….....………..26

Figure S24. IR spectrum of compound **3** in MeOH…………………………….…....………..27

Figure S25. UV spectrum of compound **3** in MeOH………………………………………..28

Figure S26. ^{1}H NMR (500 MHz) spectrum of compound **3** in MeOD…………………….………29

Figure S27. ^{13}C NMR (125 MHz) spectrum of compound **3** in MeOD………………....………30

Figure S28. ^{1}H-^{1}H COSY spectrum of compound **3** in MeOD………………………...………...31

Figure S29. HSQC spectrum of compound **3** in MeOD……………………………...………..32

Figure S30. HMBC spectrum of compound **3** in MeOD………………………...…...………..33

Figure S31. The enlarged HMBC spectrum of compound 3 in 0.6~1.8 ppm in MeOD.....…..…..34

Figure S32. NOESY spectrum of compound **3** in MeOD…………………………………...35

Figure S33. TOCSY spectrum of compound **3** in MeOD……………………………………36

Figure S34. The structures of compounds **A1–A19**…………………………………………37

Table S1. Binding affinity of different compounds to inflammatory pathway related target proteins………………………………………………………………………………………38

Table S2. The optimized lowest energy 3D conformers and energy analysis of compounds **1a–3a**……………….………………….………………….…………………………….…...39

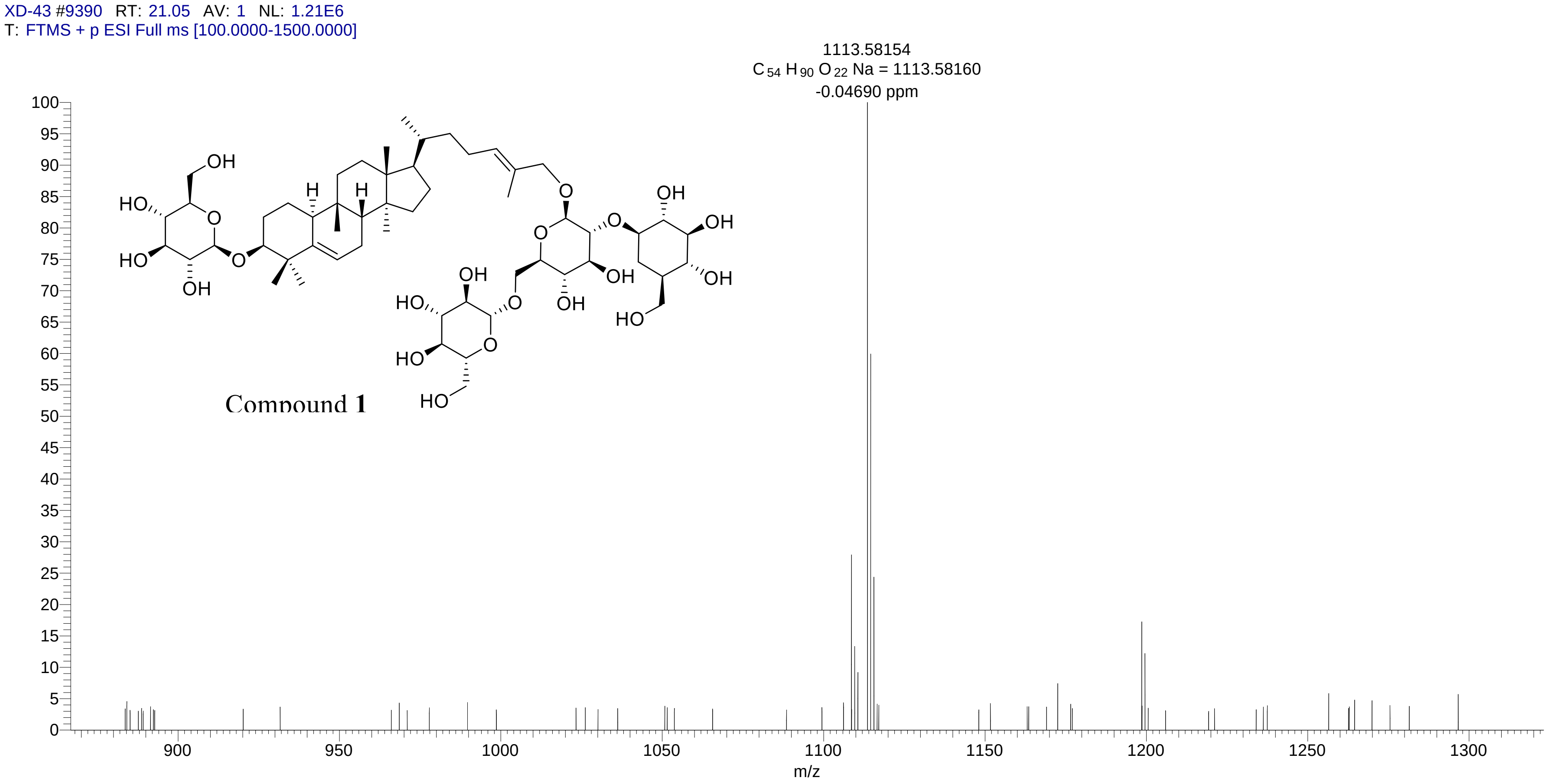

Figure S1. HR-ESI-MS spectrum of compound **1** in MeOH.

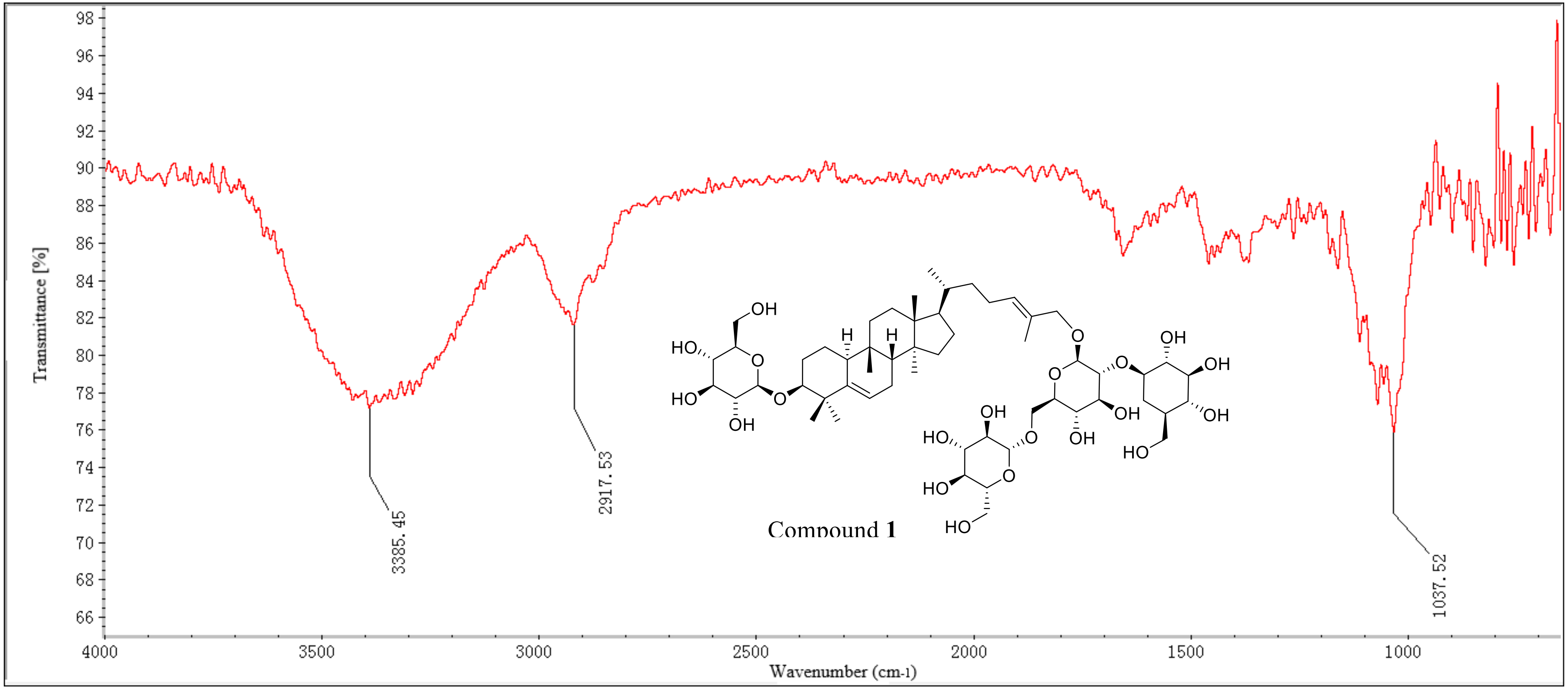

Figure S2. IR spectrum of compound **1** in MeOH.

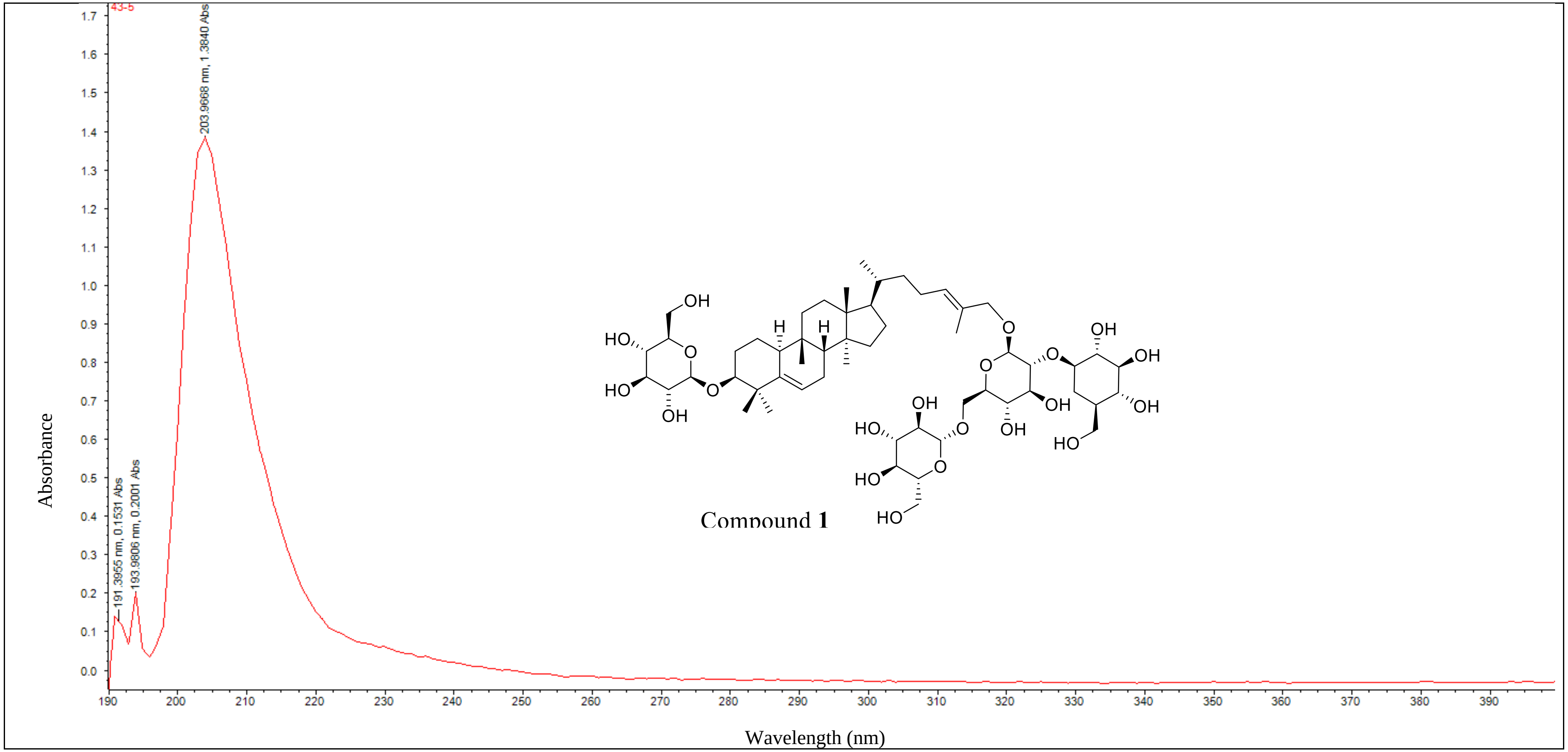

Figure S3. UV spectrum of compound **1** in MeOH.

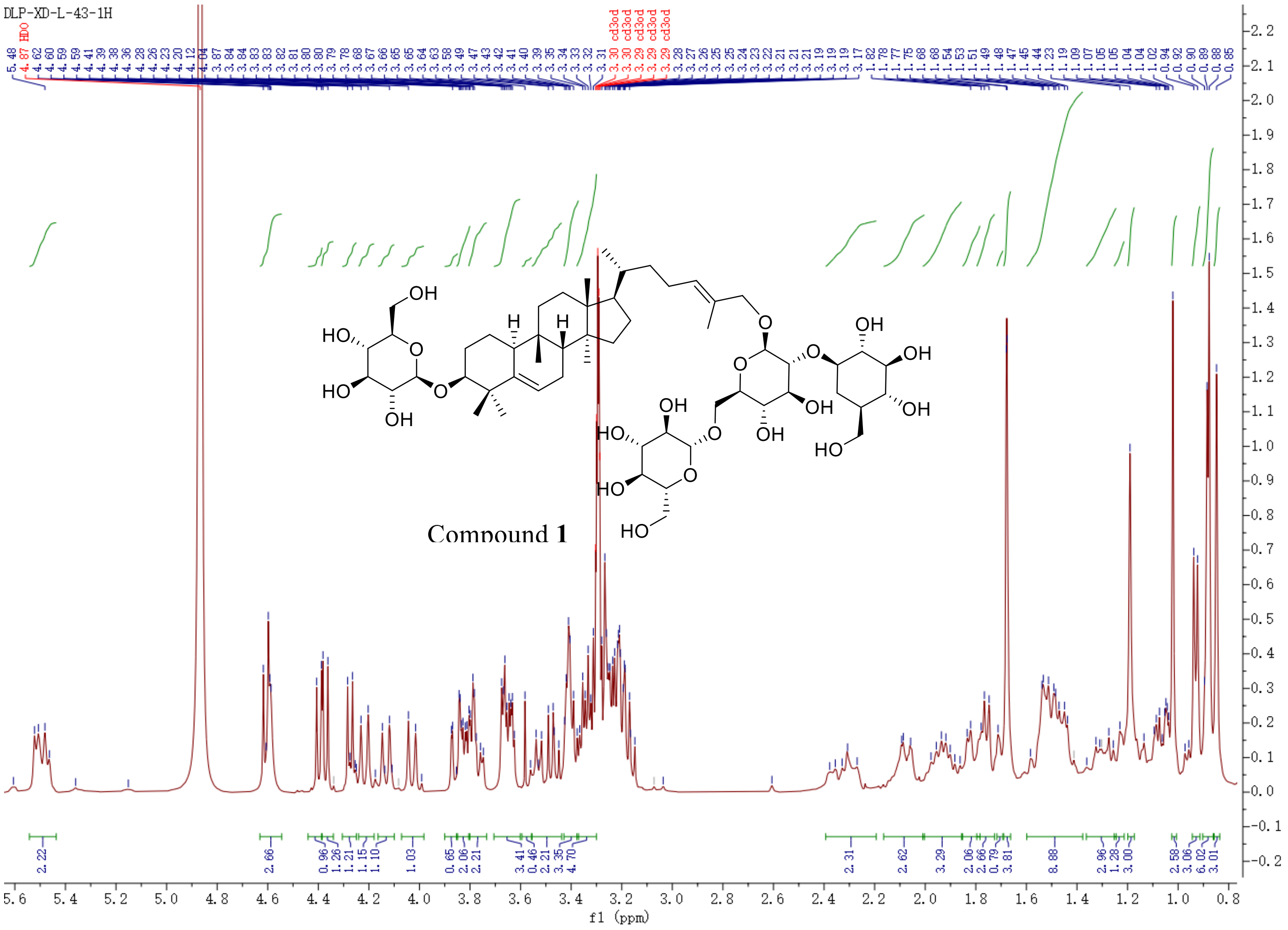

Figure S4. ^{1}H NMR (500 MHz) spectrum of compound **1** in MeOD.

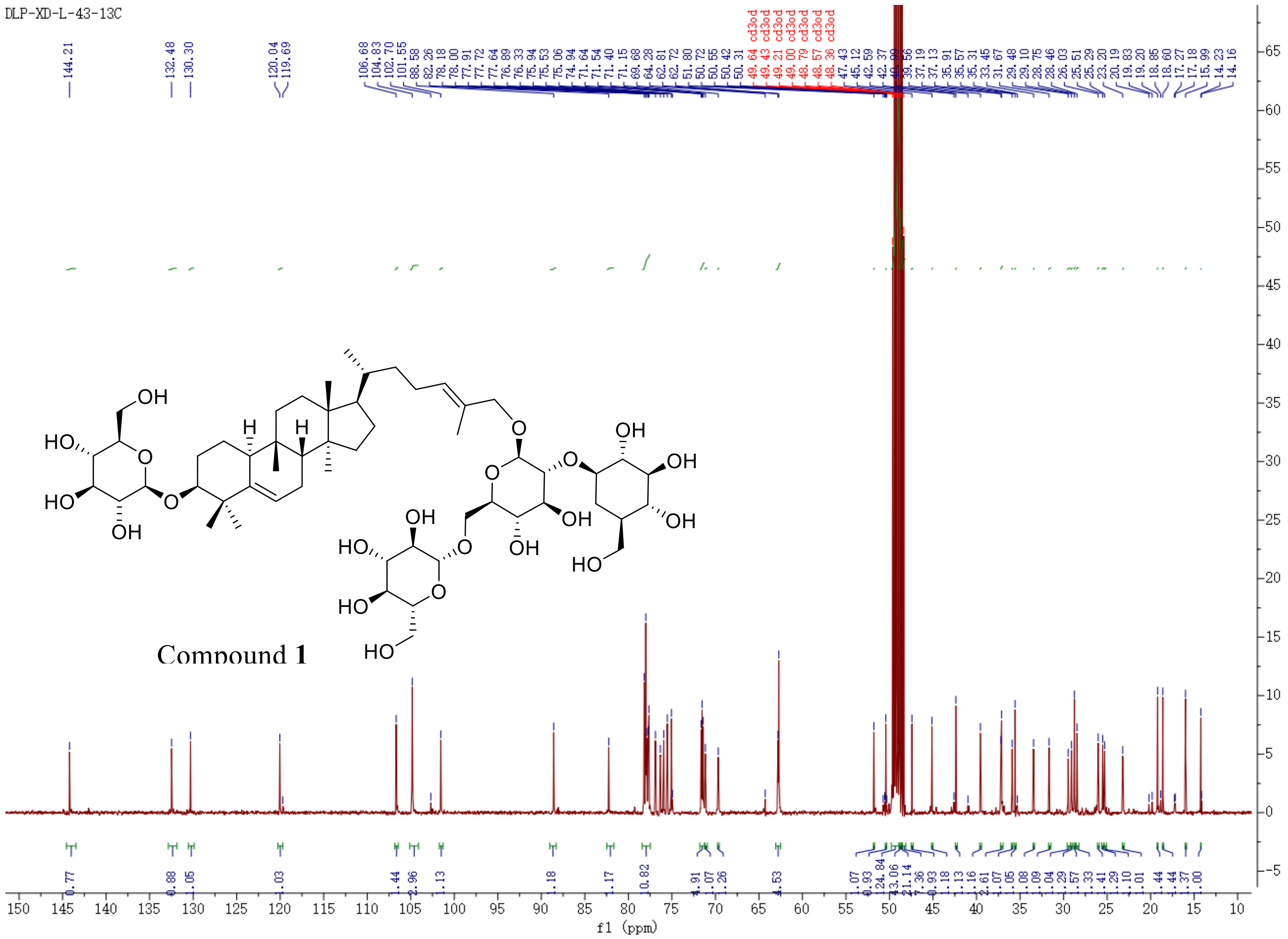

Figure S5. ^{13}C NMR (125 MHz) spectrum of compound **1** in MeOD.

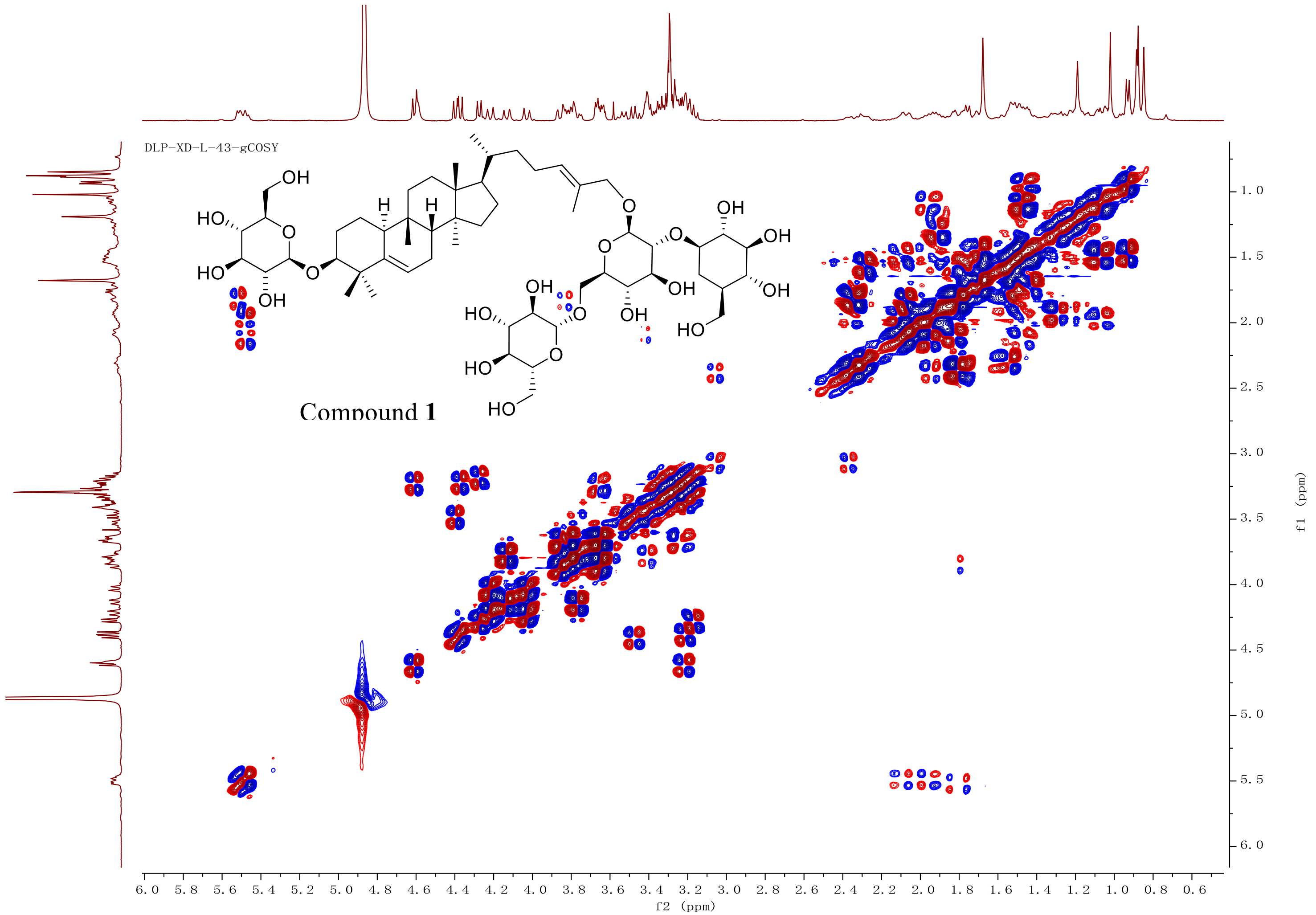

Figure S6. gCOSY spectrum of compound **1** in MeOD.

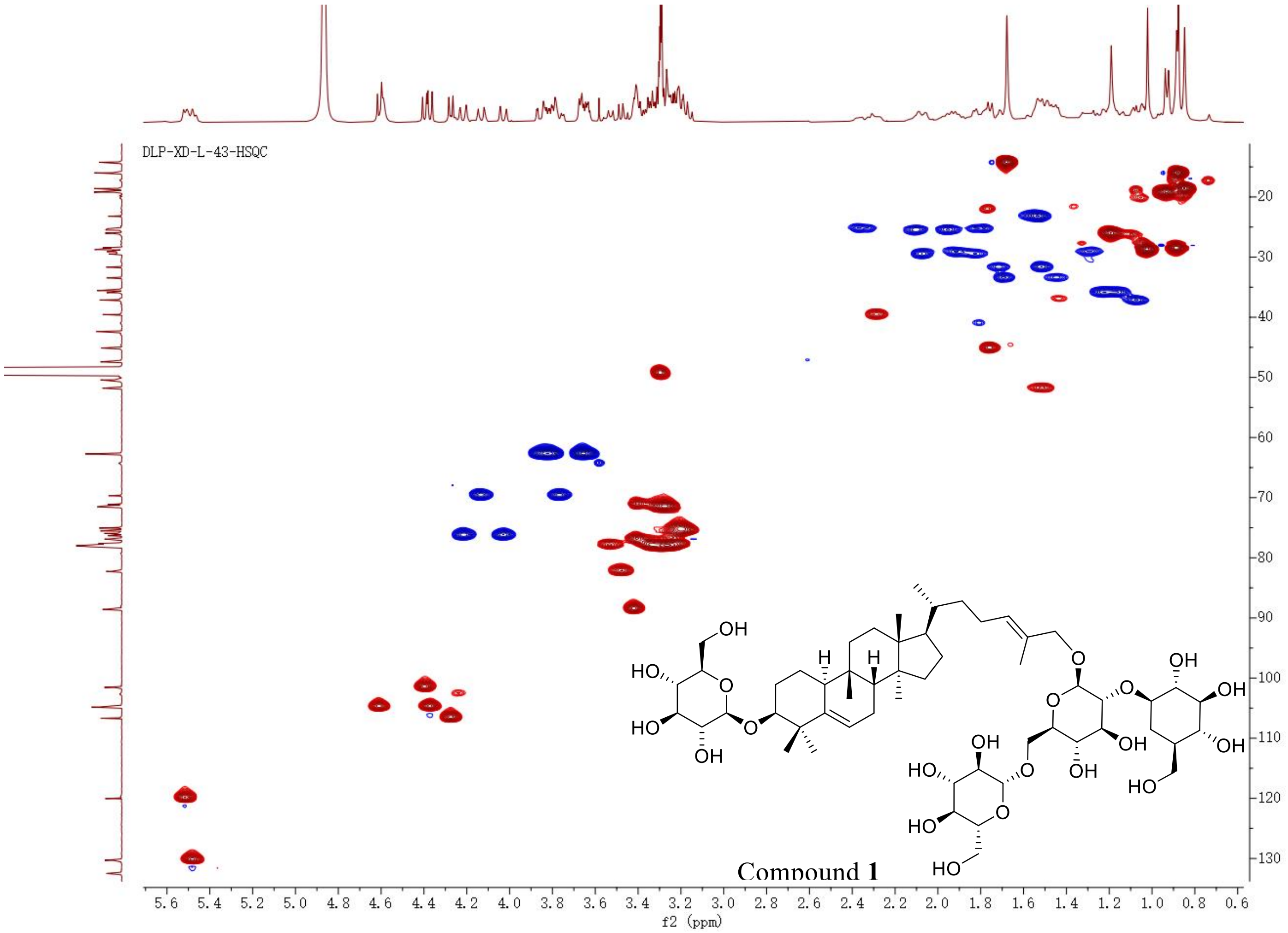

Figure S7. HSQC spectrum of compound **1** in MeOD.

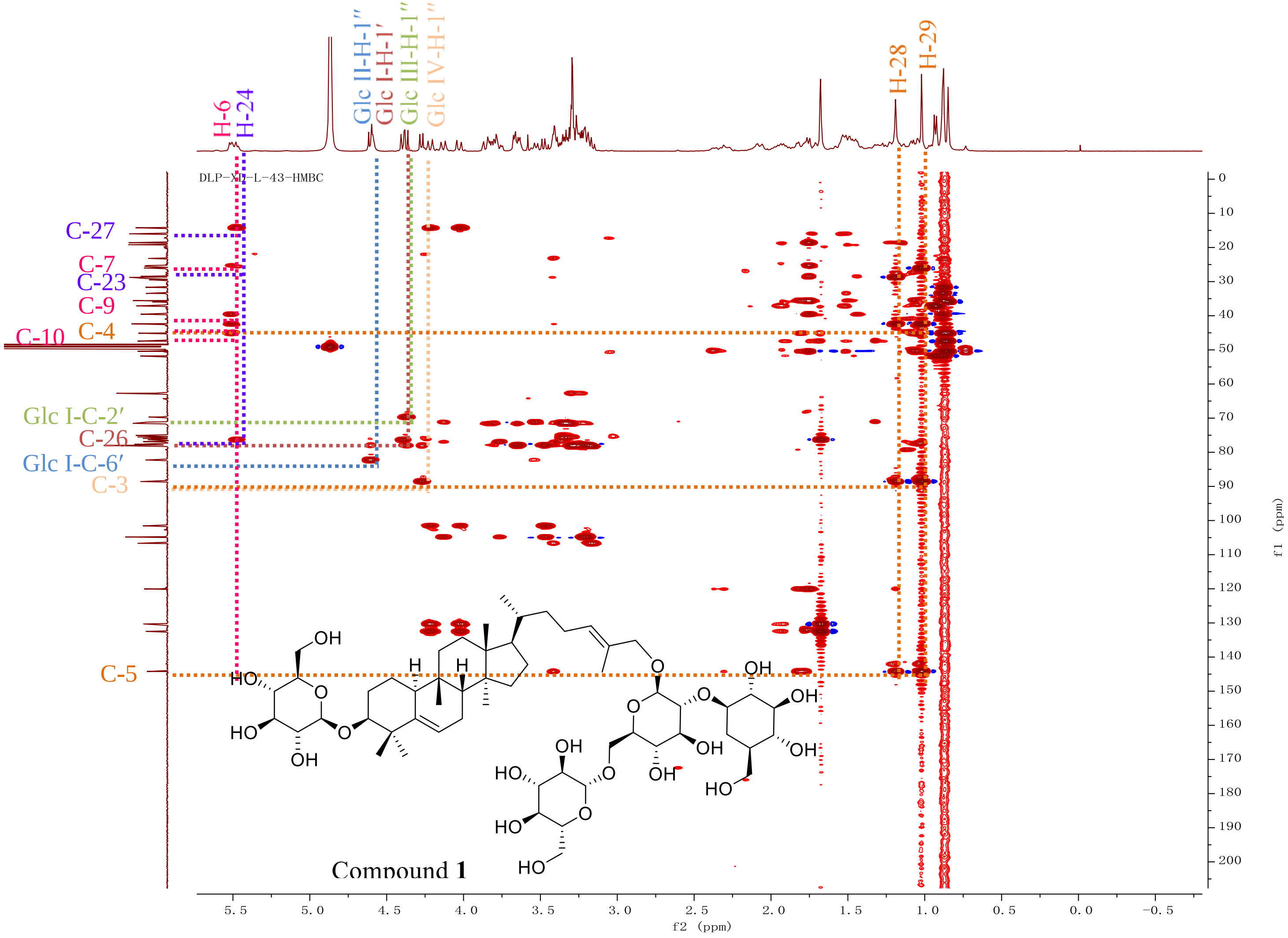

Figure S8. HMBC spectrum of compound **1** in MeOD.

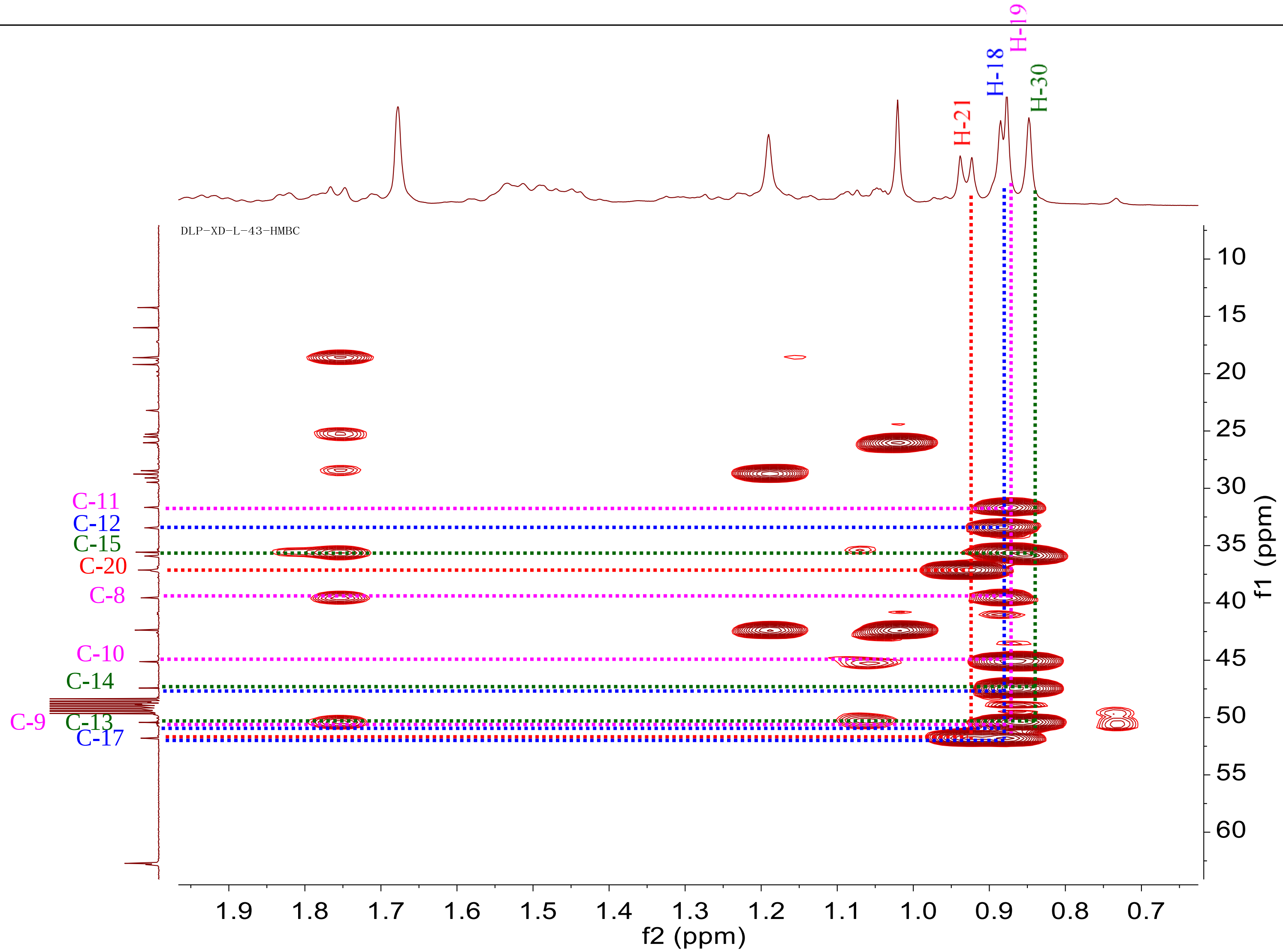

Figure S9. The enlarged HMBC spectrum of compound **1** in 0.7~1.9 ppm in MeOD.

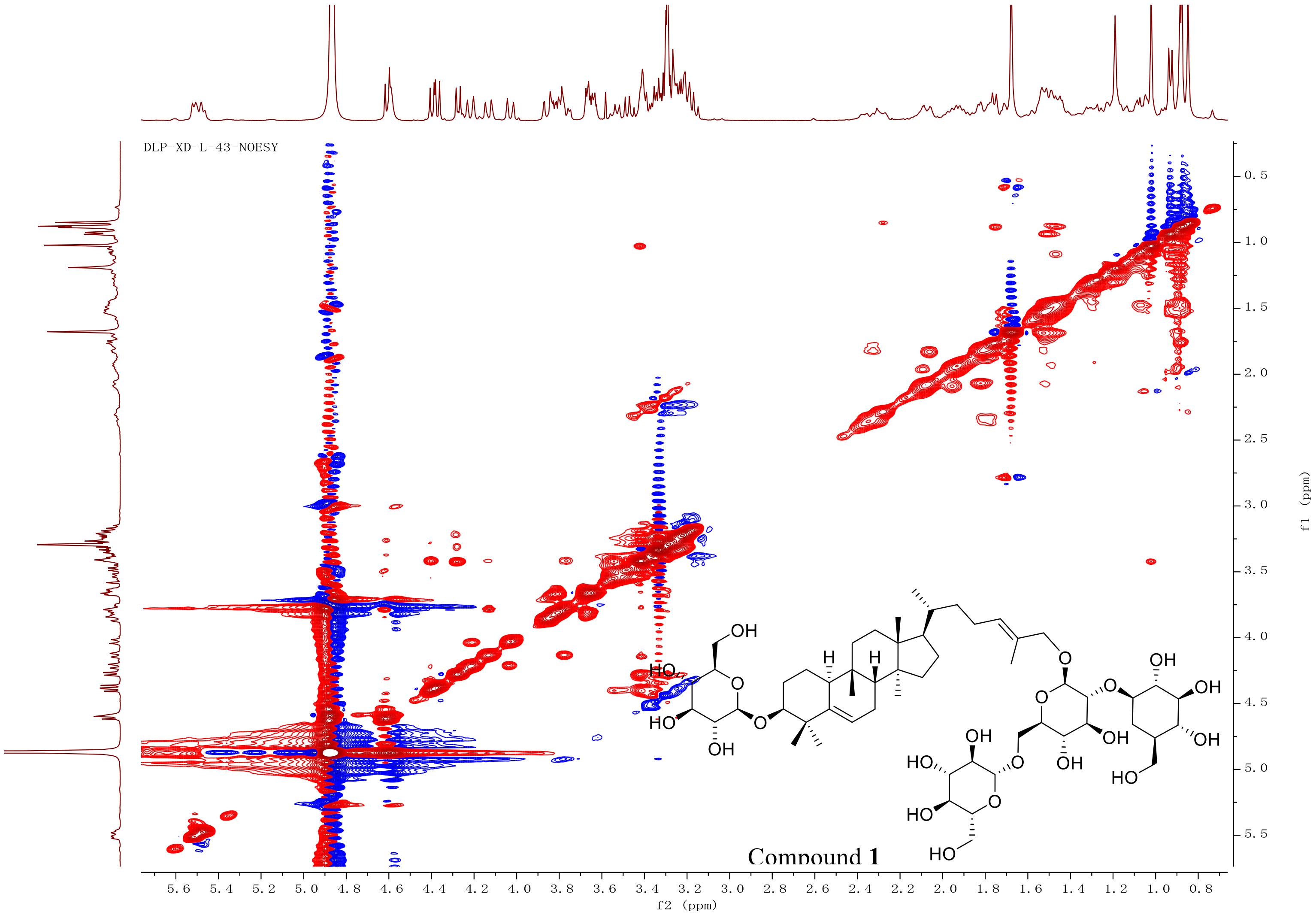

Figure S10. NOESY spectrum of compound **1** in MeOD.

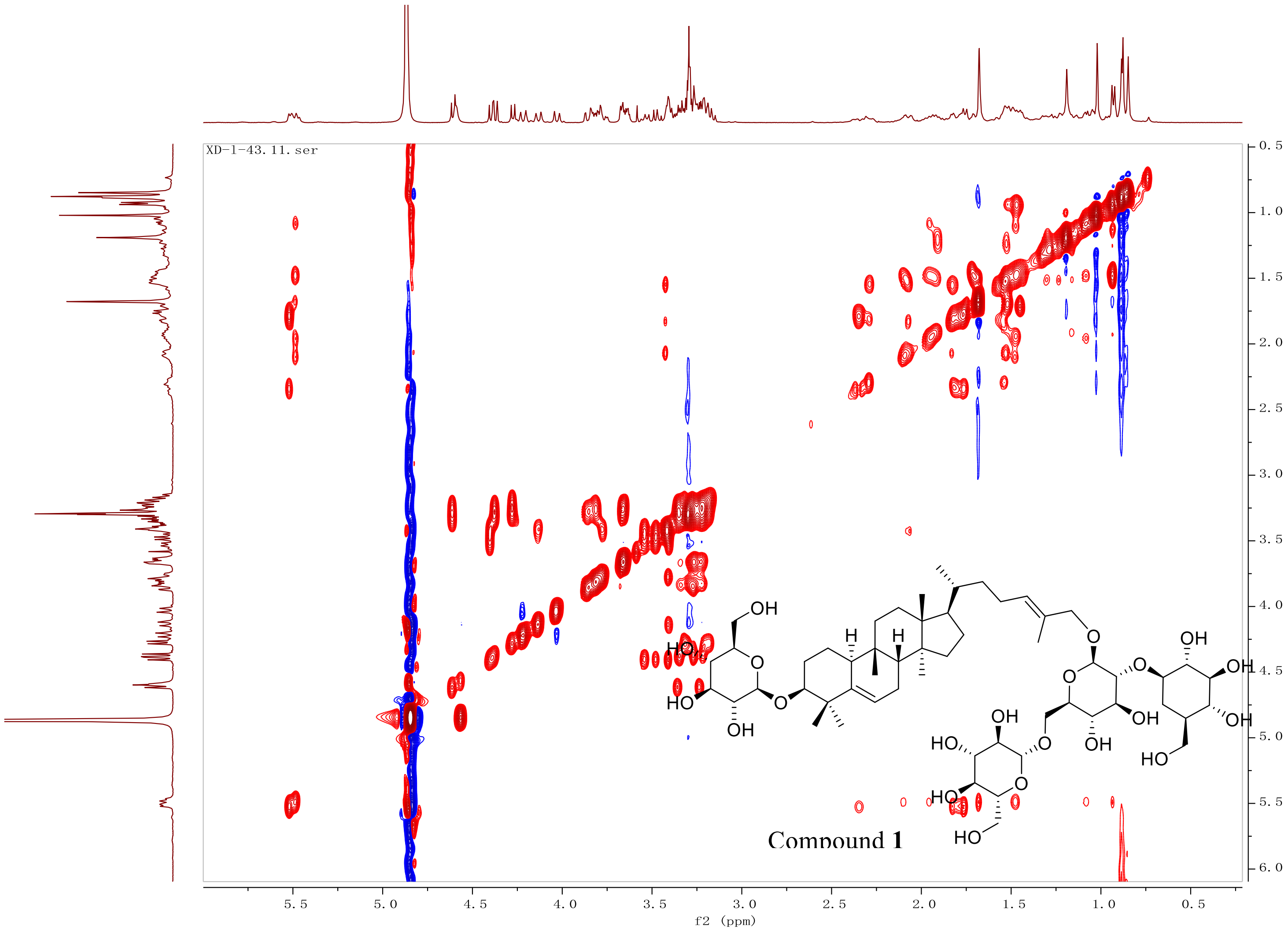

Figure S11. TOCSY spectrum of compound **1** in MeOD.

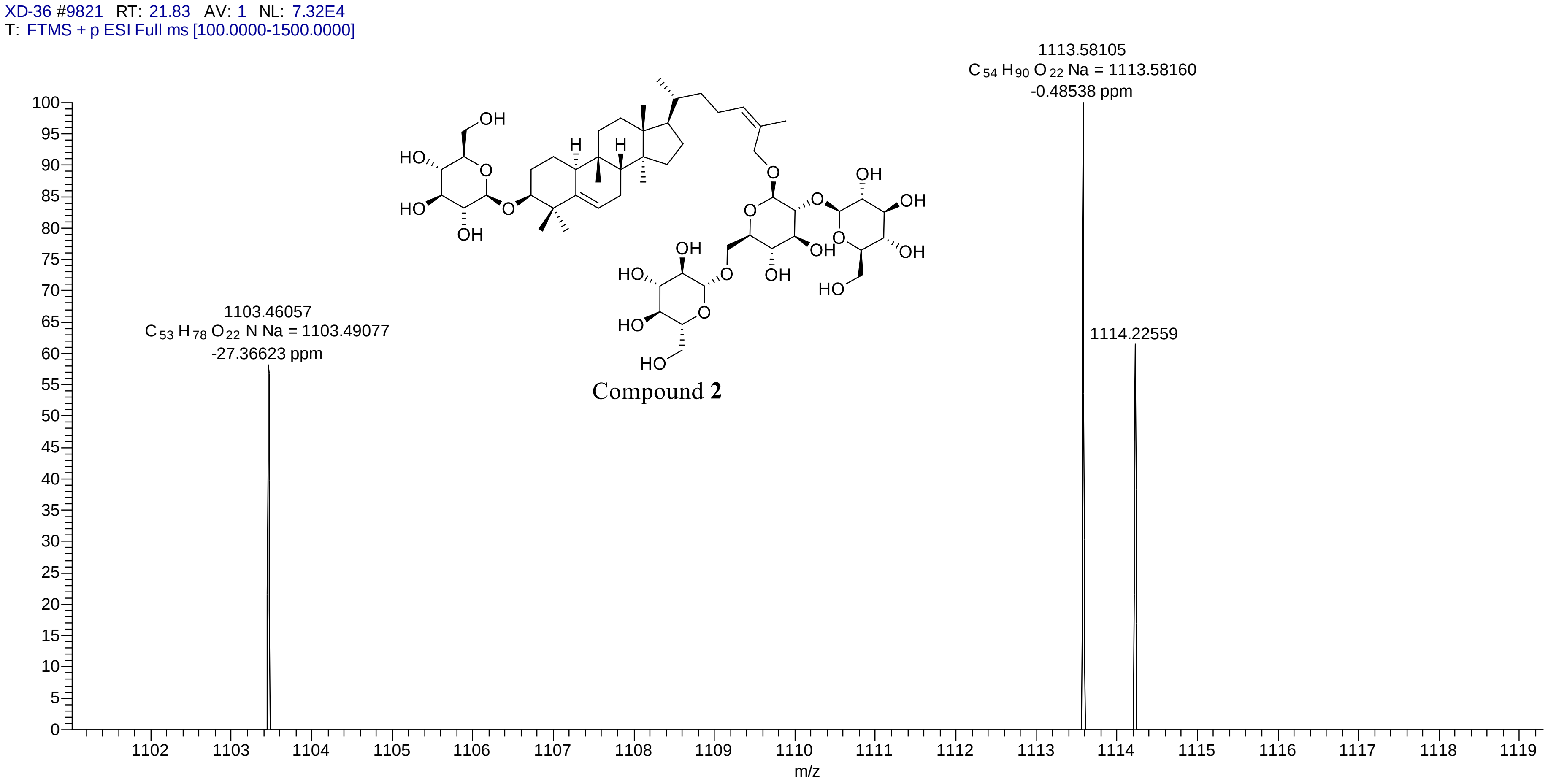

Figure S12. HR-ESI-MS spectrum of compound **2** in MeOH.

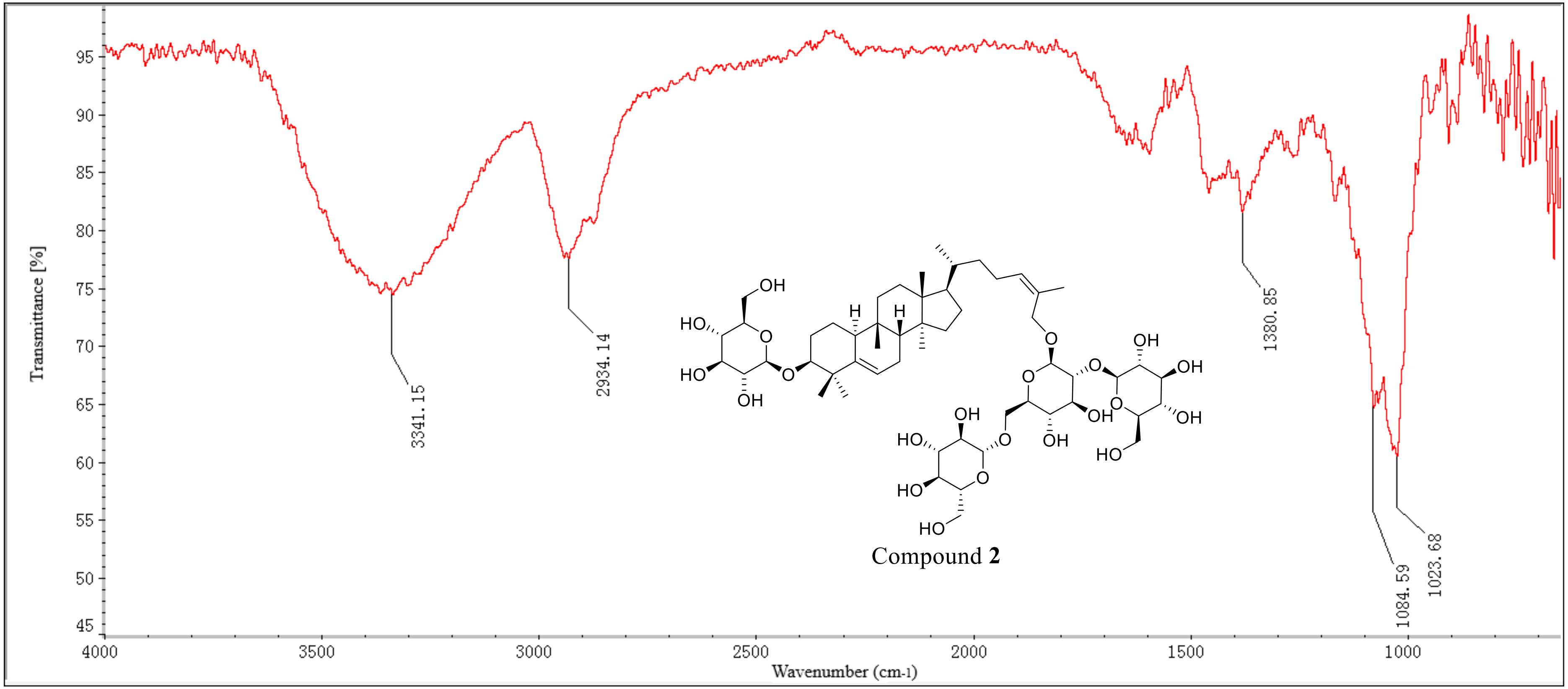

Figure S13. IR spectrum of compound **2** in MeOH.

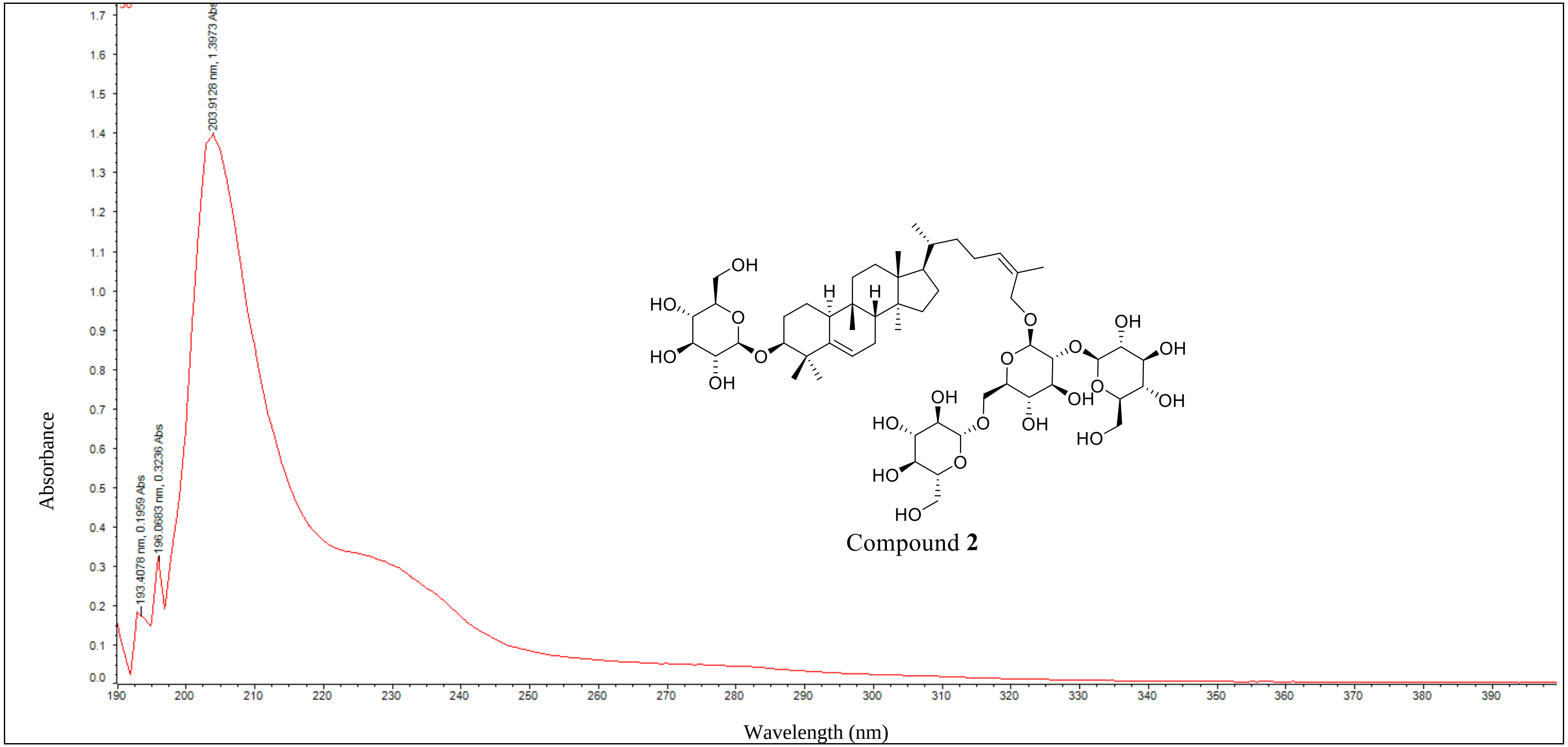

Figure S14. UV spectrum of compound **2** in MeOH.

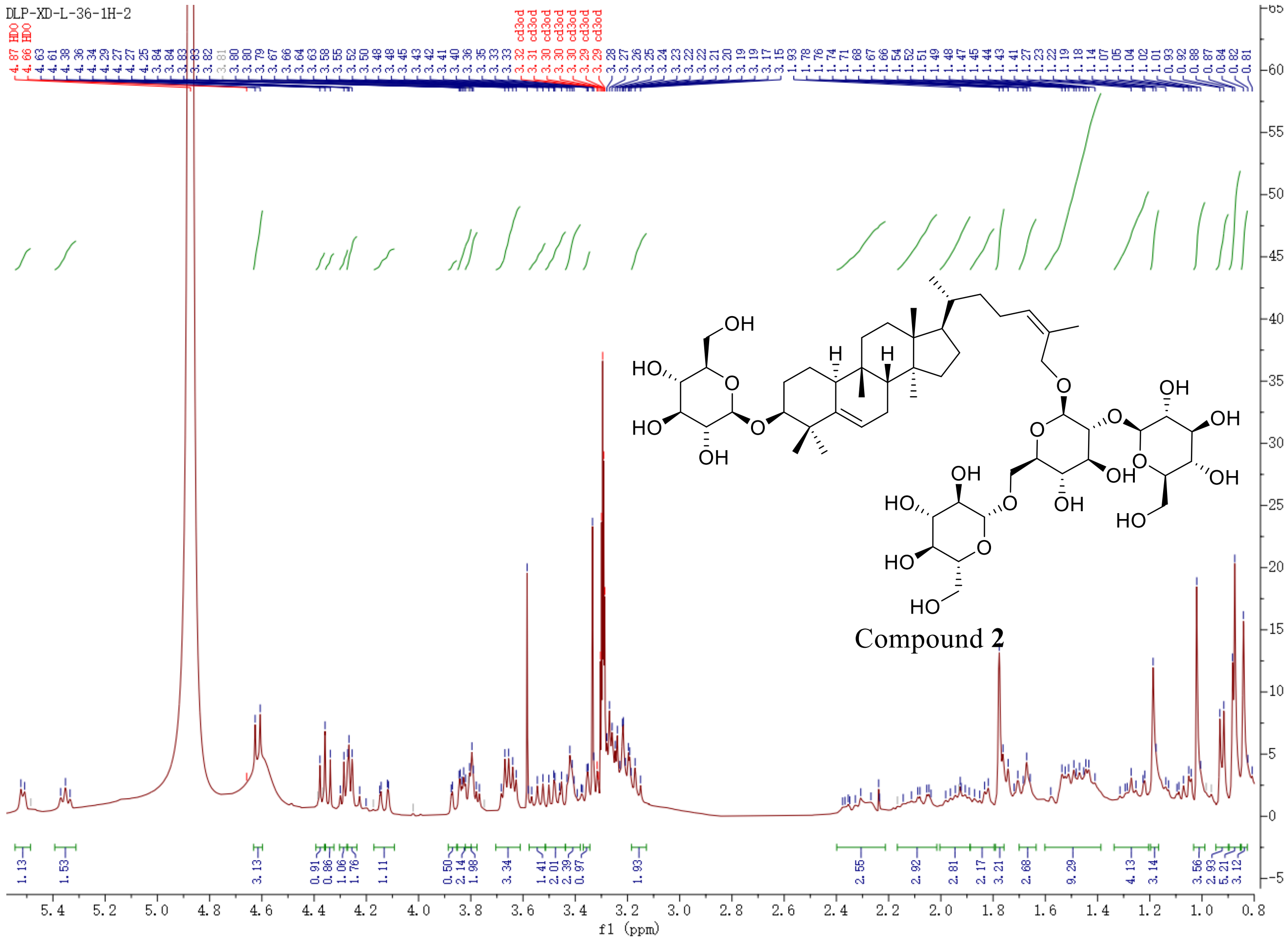

Figure S15.^{1}HNMR (500 MHz) spectrum of compound **2** in MeOD.

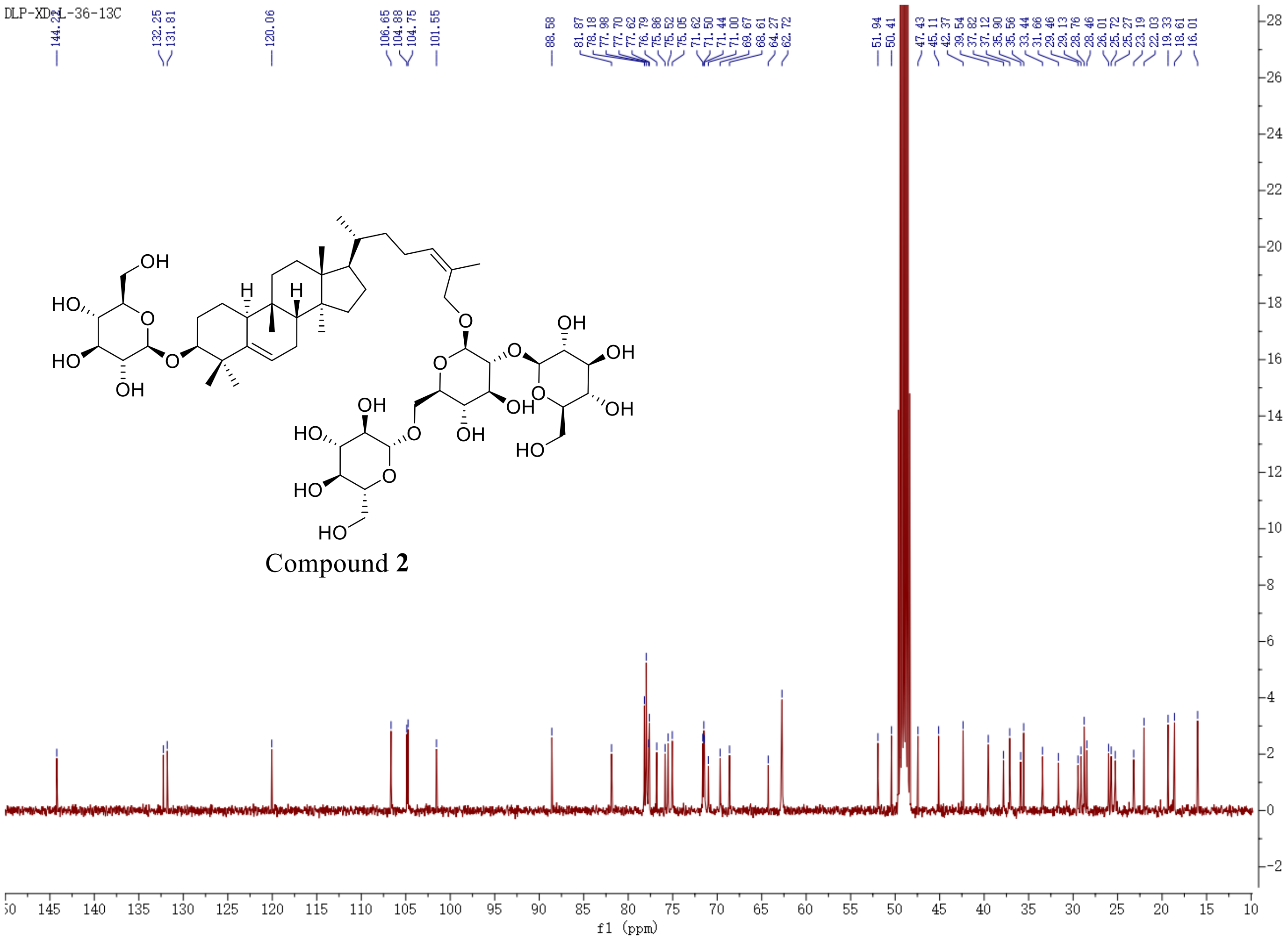

Figure S16. ¹³CNMR (125 MHz) spectrum of compound **2** in MeOD.

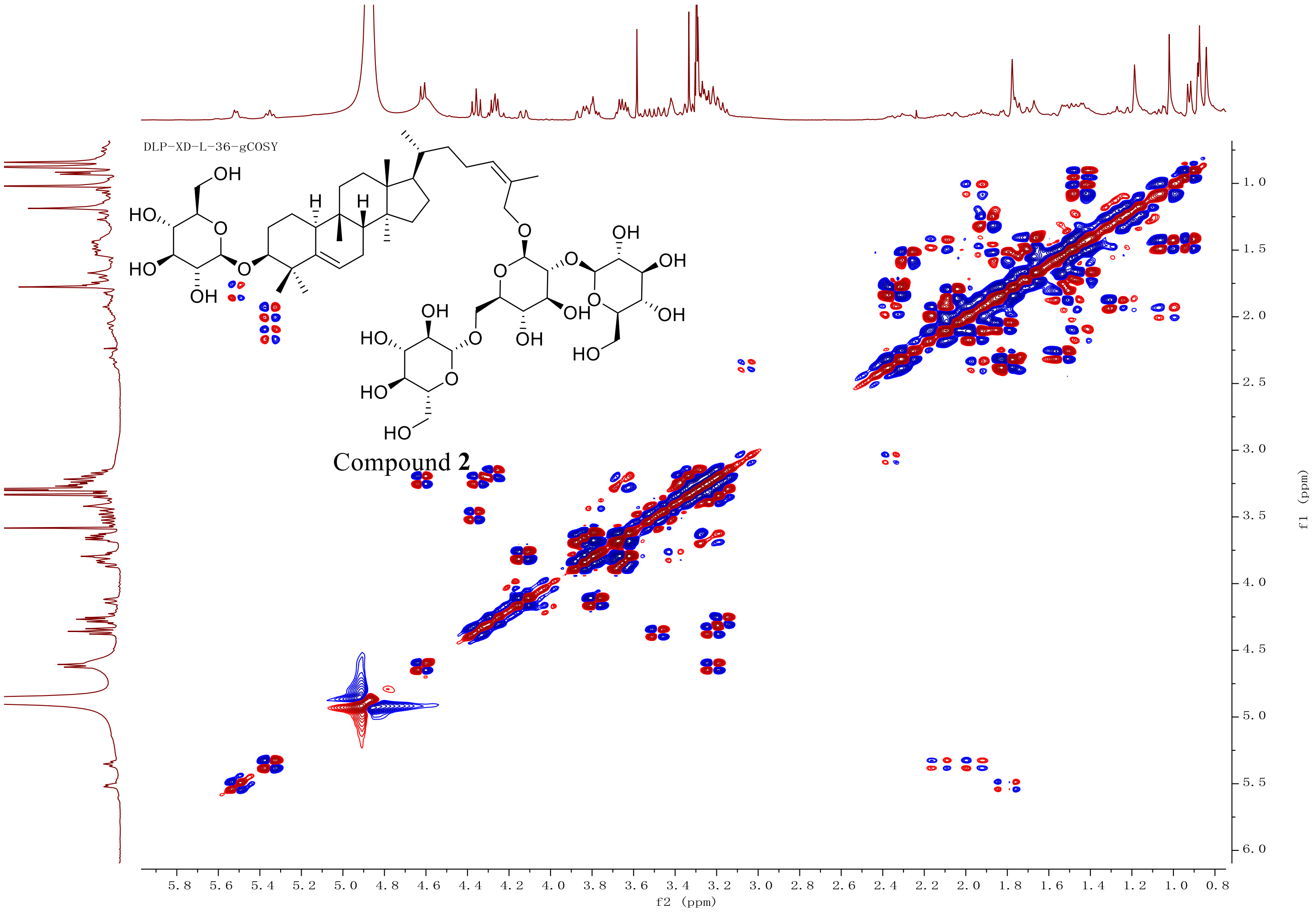

Figure S17. gCOSY spectrum of compound **2** in MeOD.

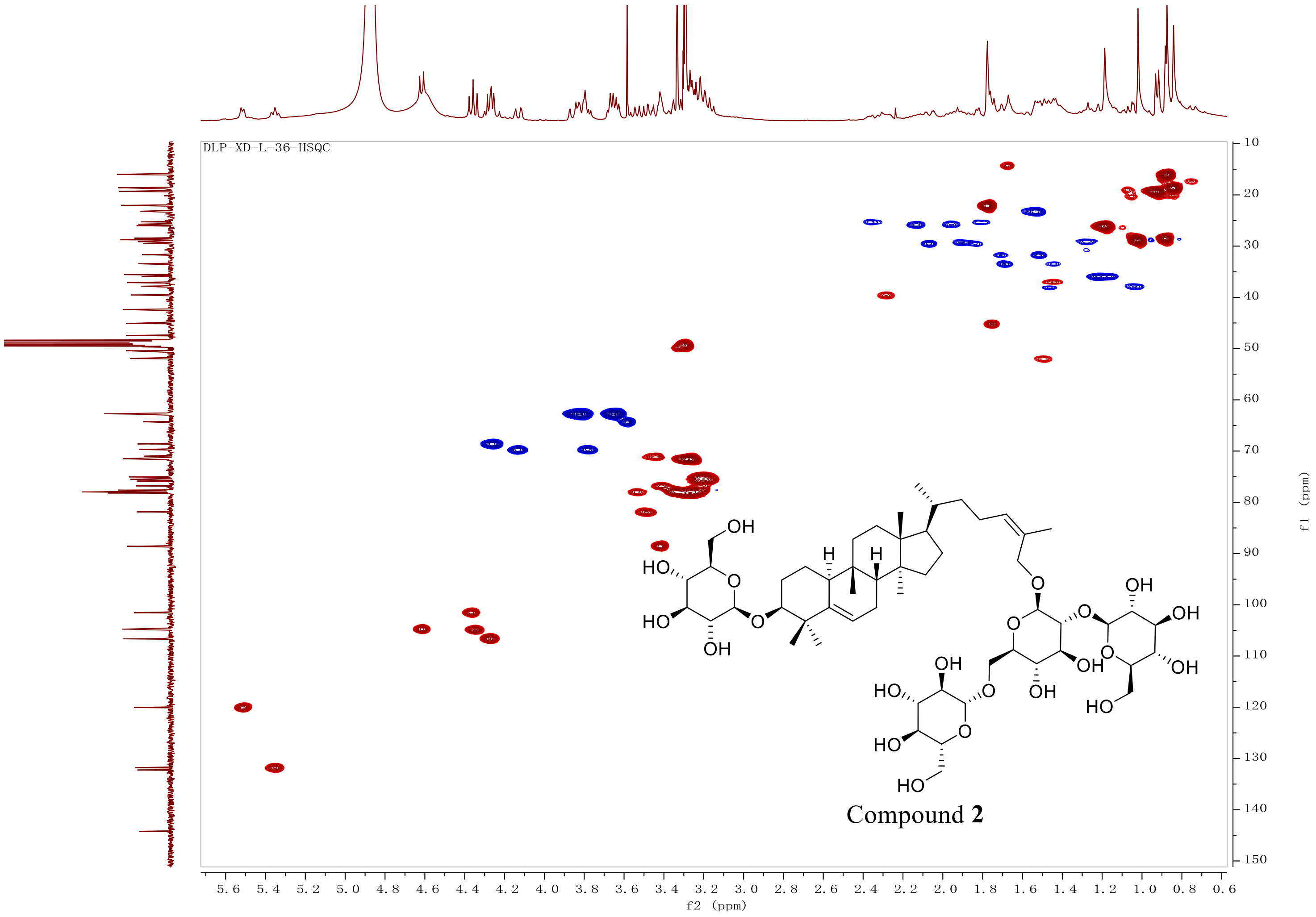

Figure S18. HSQC spectrum of compound **2** in MeOD.

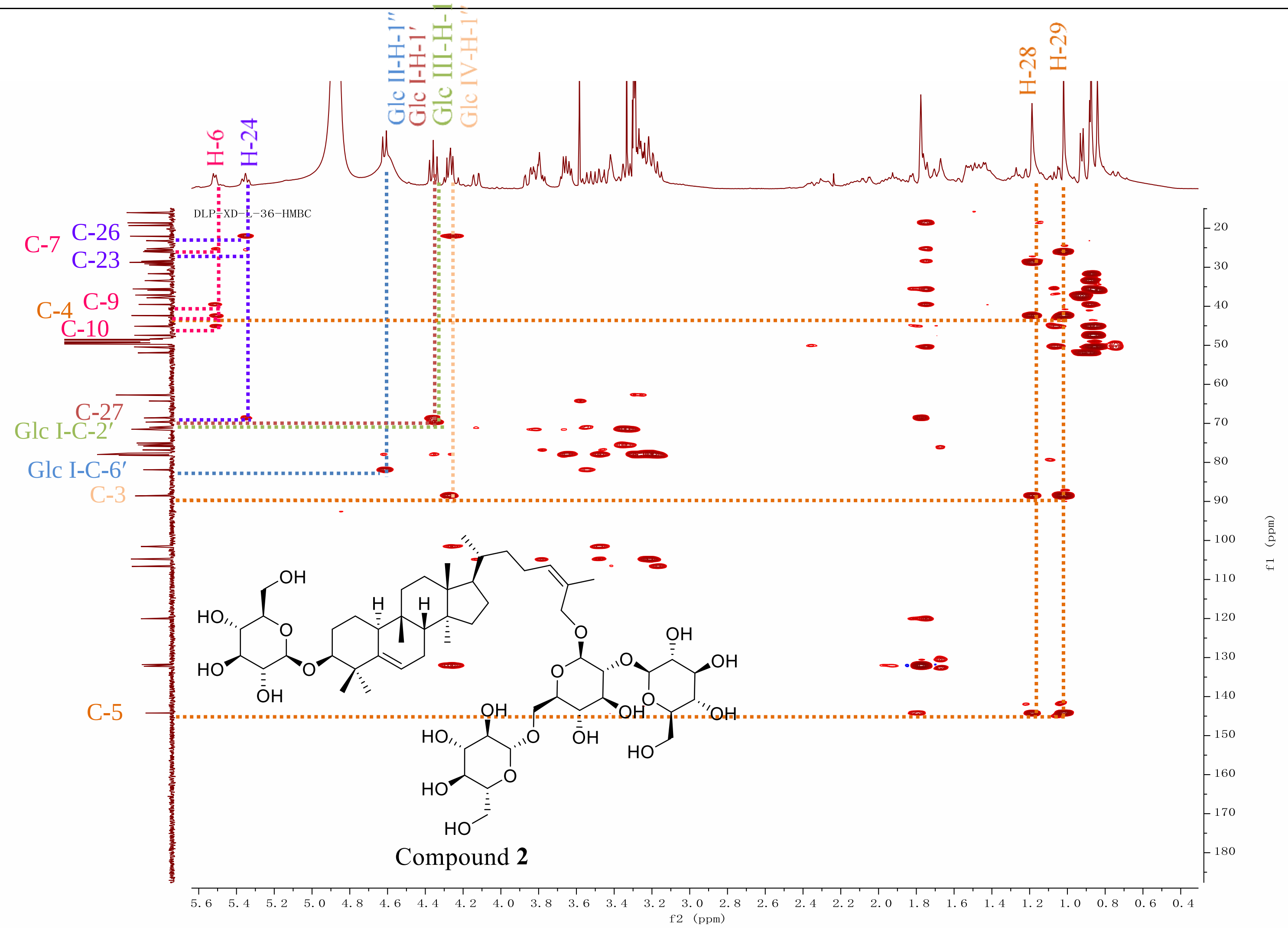

Figure S19. HMBC spectrum of compound **2** in MeOD.

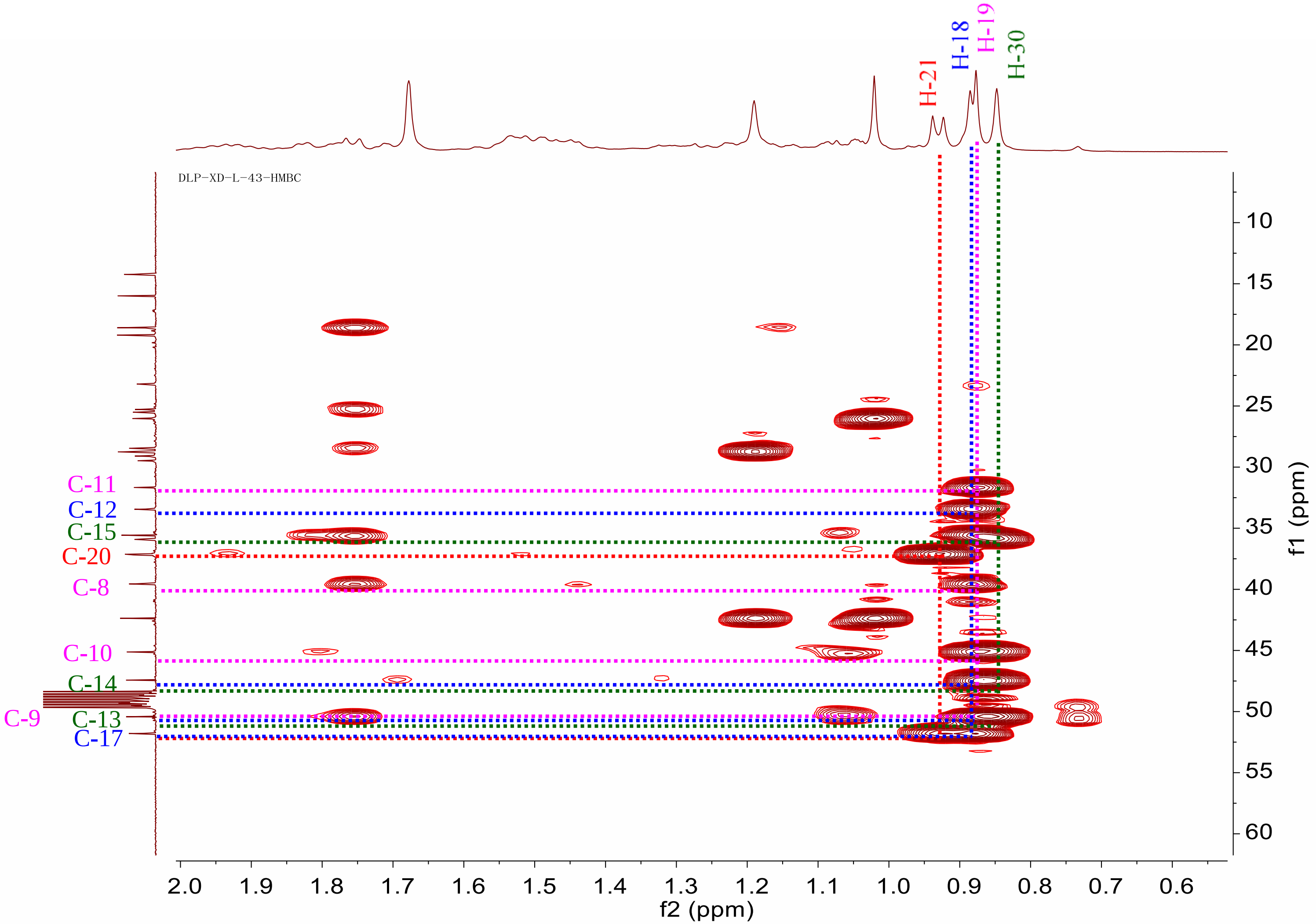

Figure S20. The enlarged HMBC spectrum of compound **2** in 0.6~2.0 ppm in MeOD.

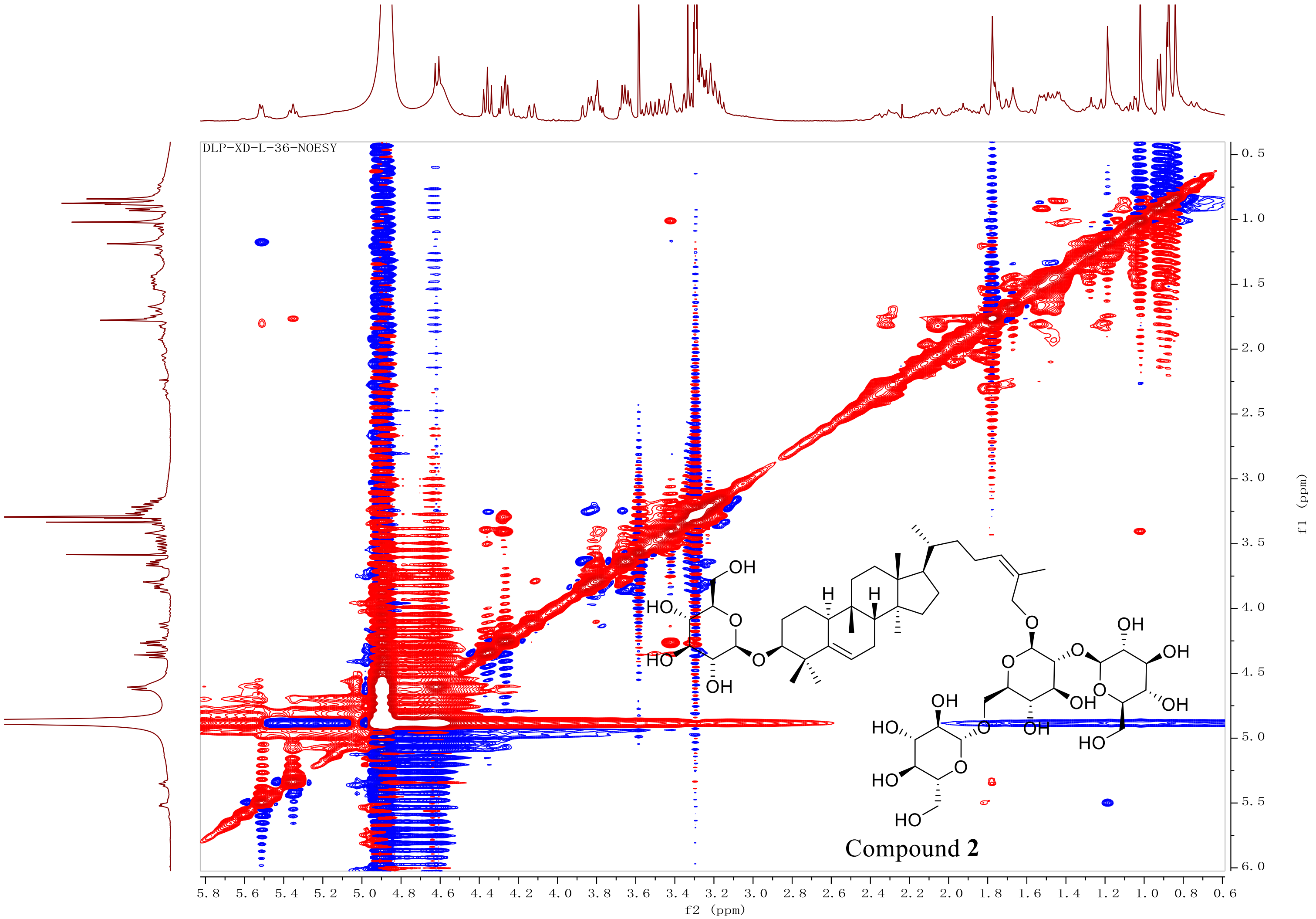

Figure S21. NOESY spectrum of compound **2** in MeOD.

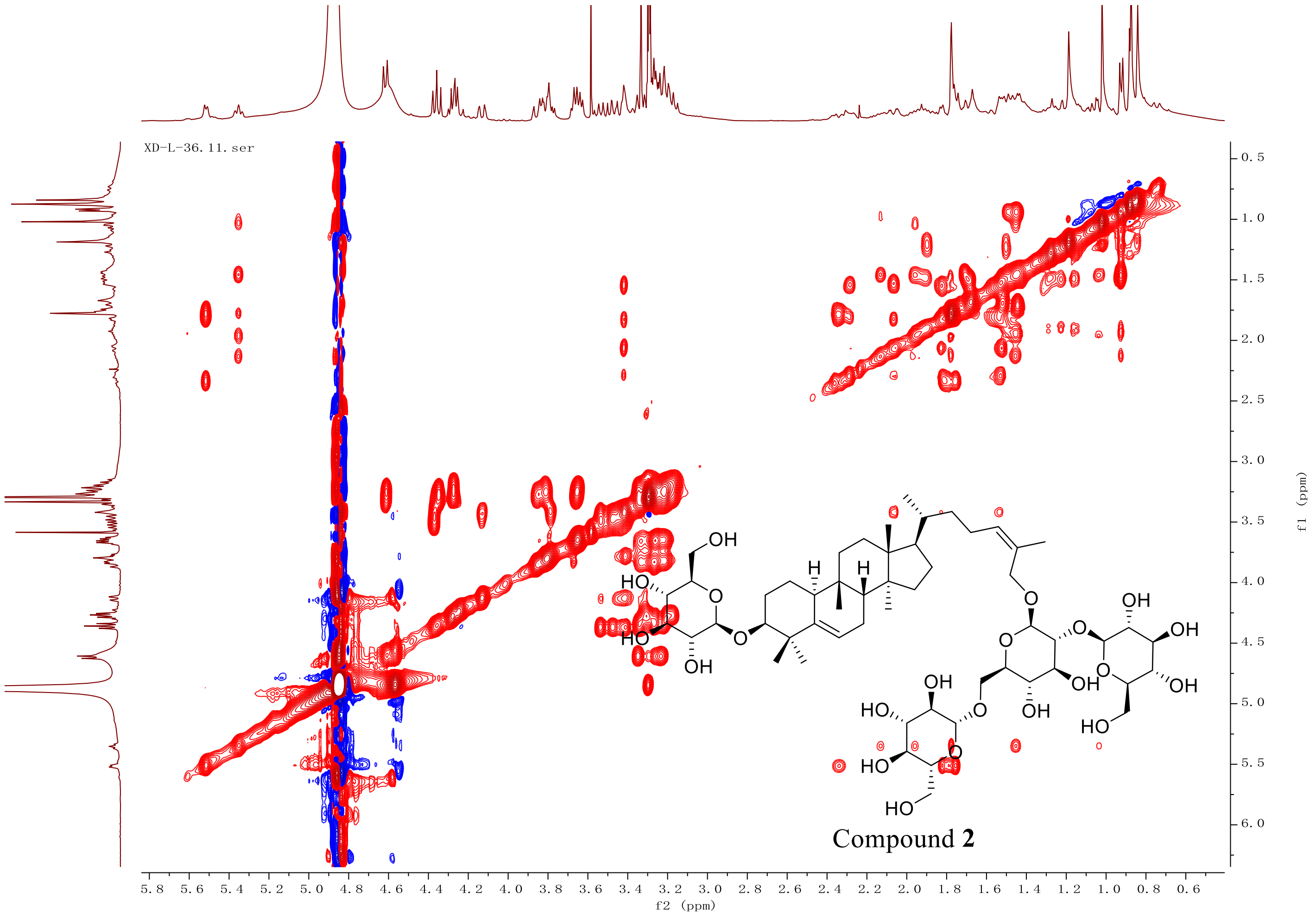

Figure S22. TOCSY spectrum of compound **2** in MeOD.

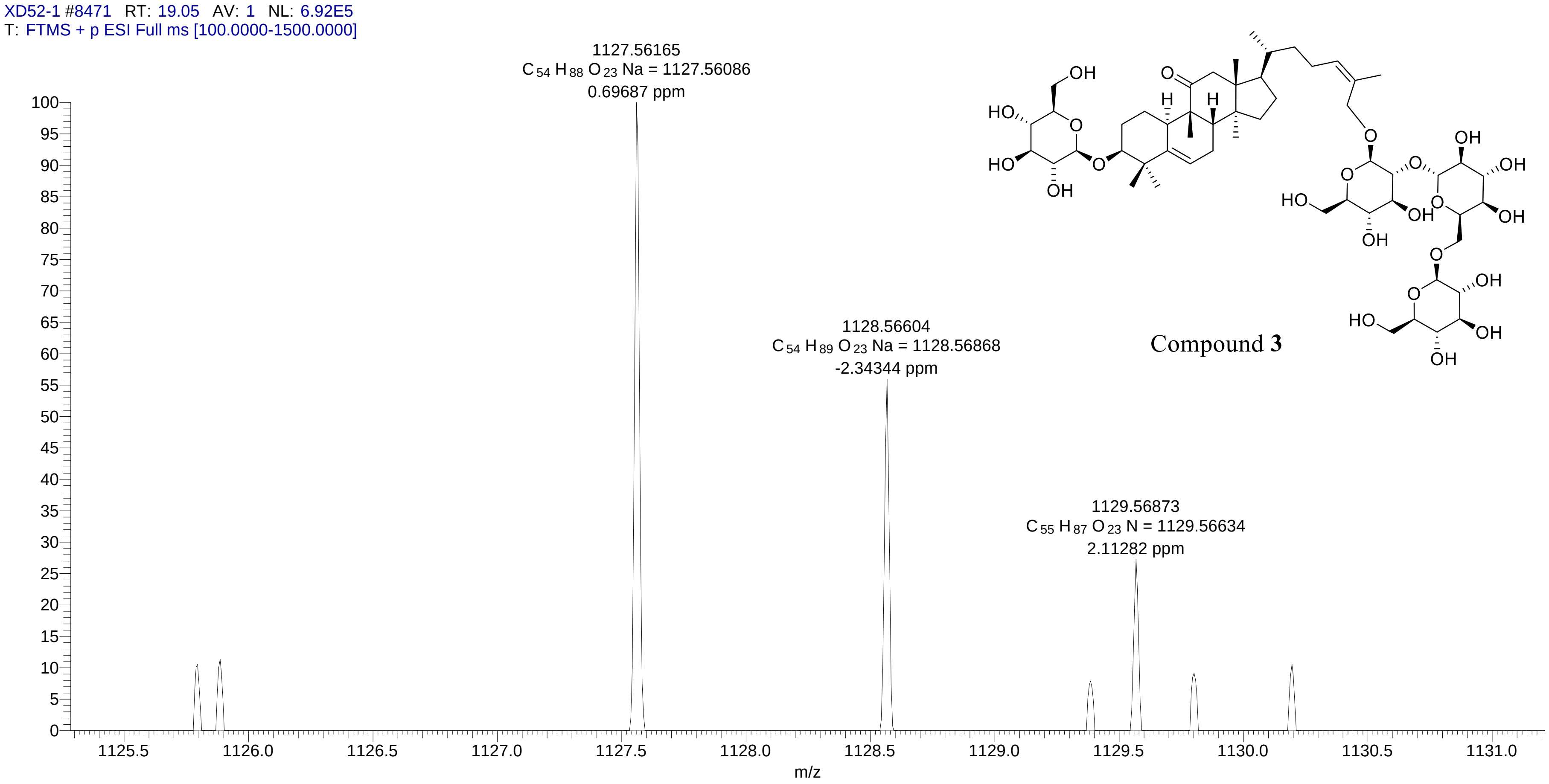

Figure S23. HR-ESI-MS spectrum of compound **3** in MeOH.

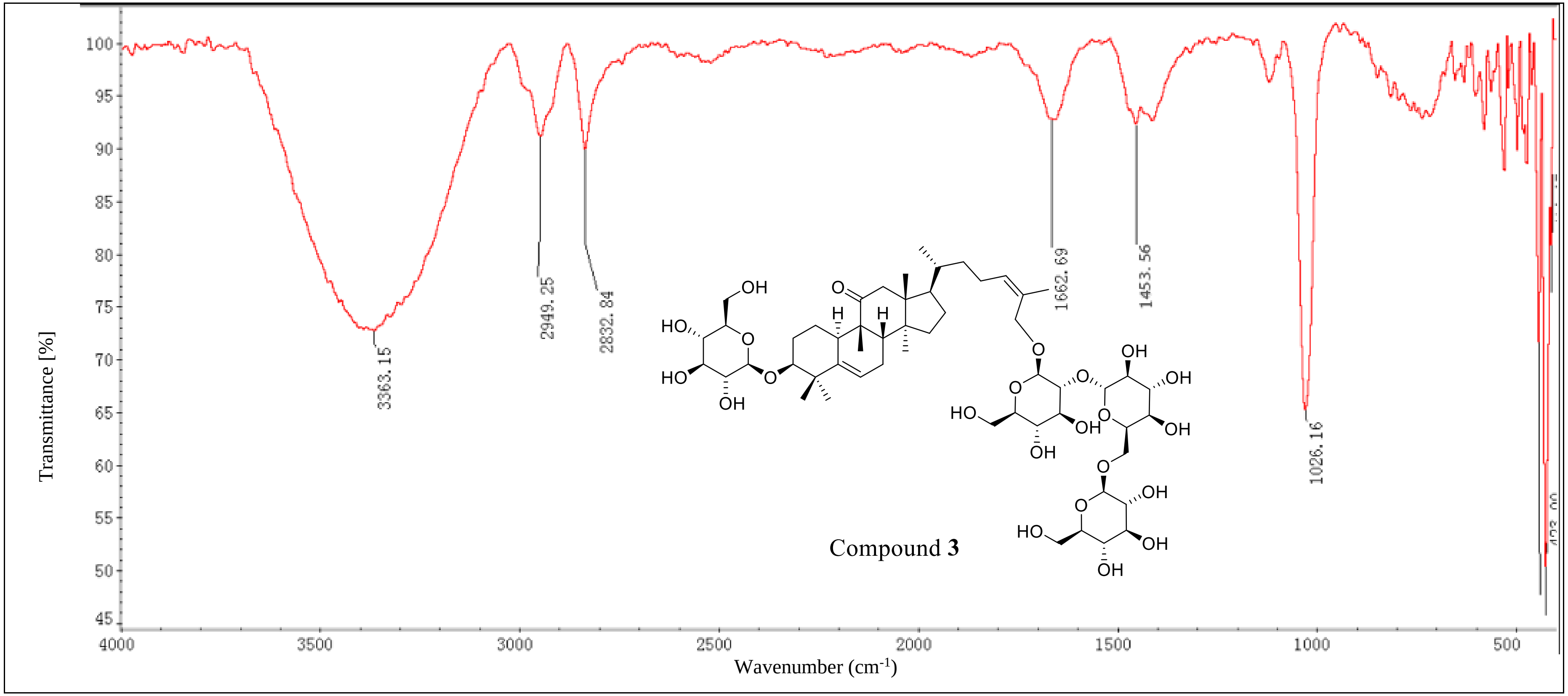

Figure S24. IR spectrum of compound **3** in MeOH.

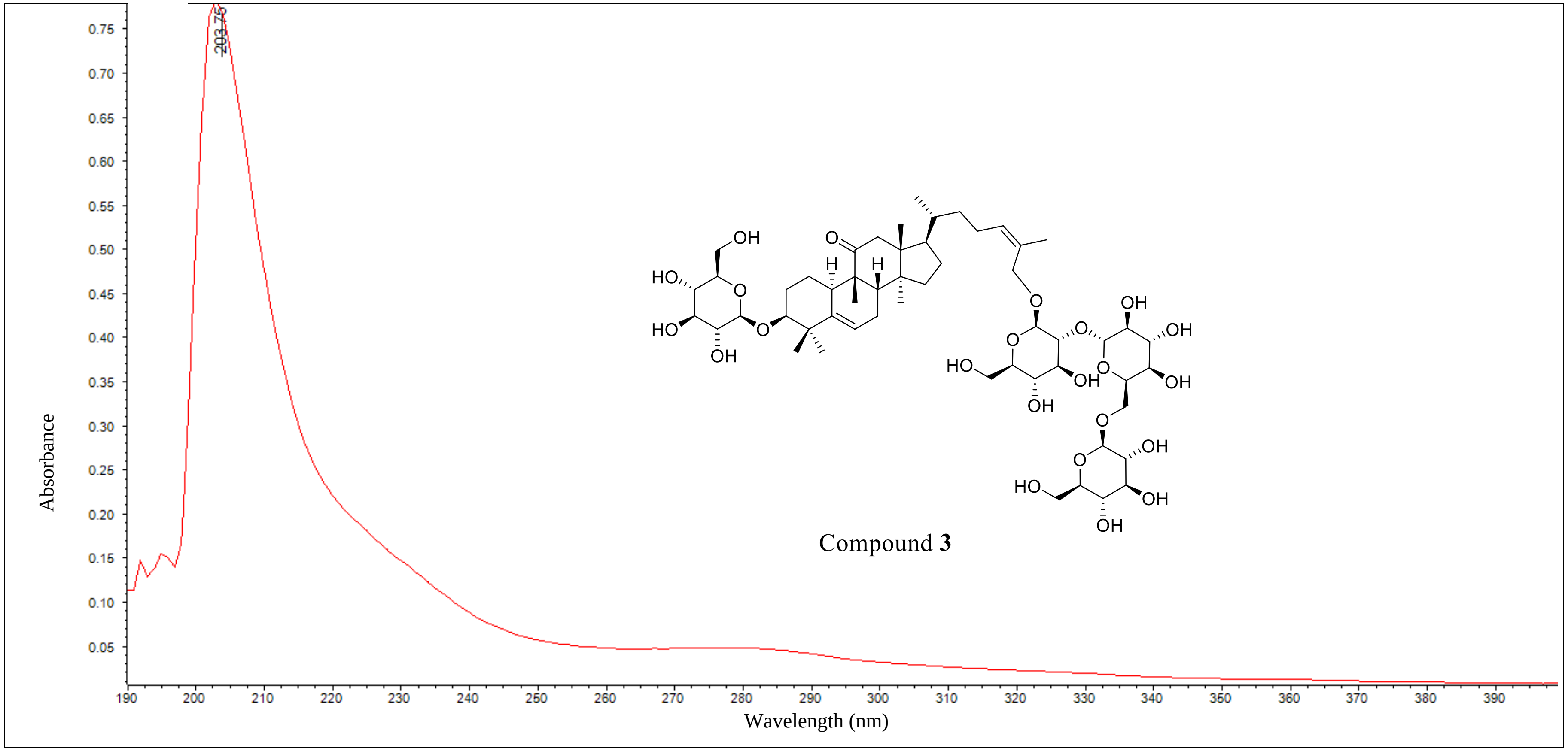

Figure S25. UV spectrum of compound **3** in MeOH.

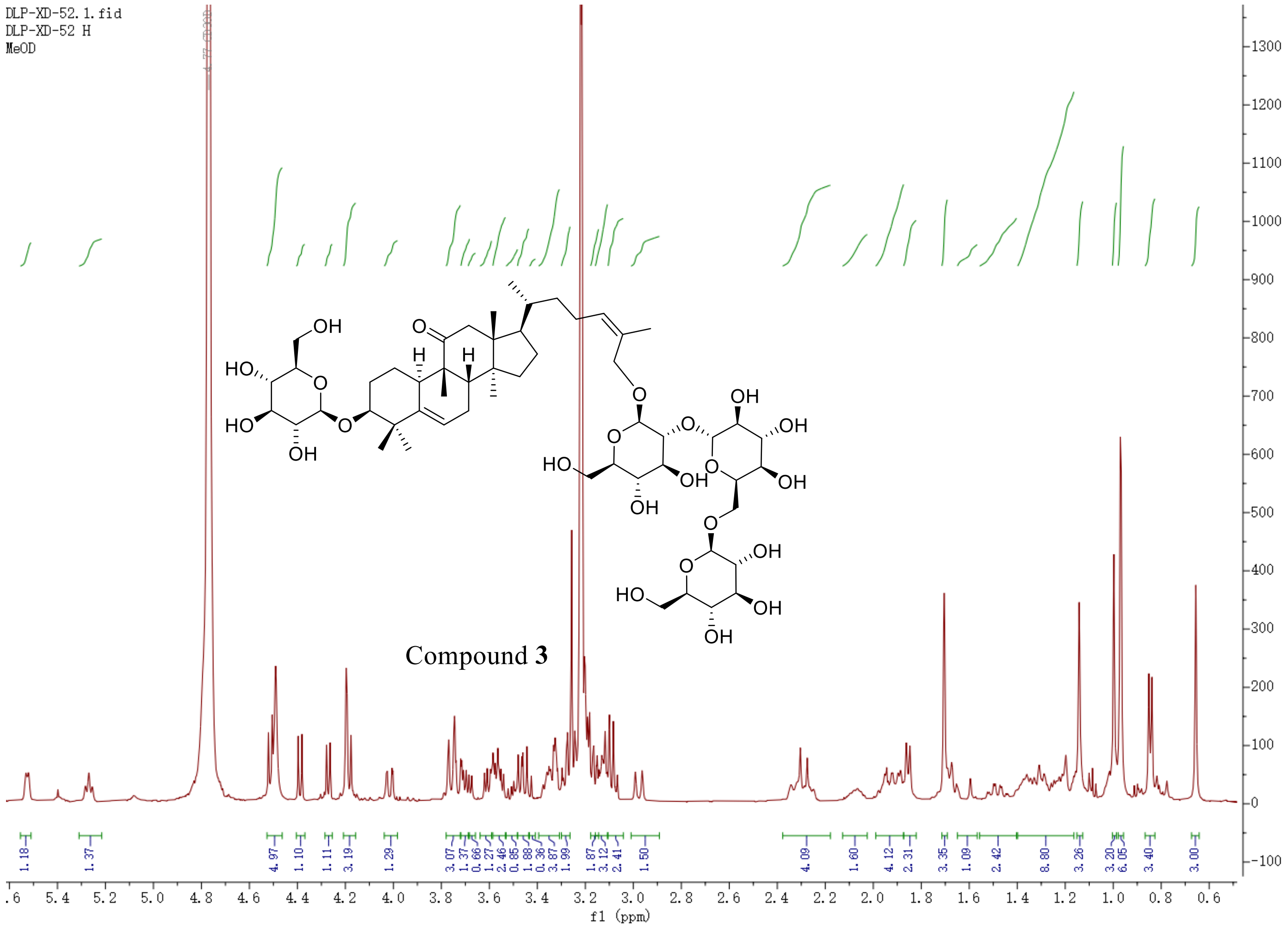

Figure S26. ^{1}HNMR (500 MHz) spectrum of compound **3** in MeOD.

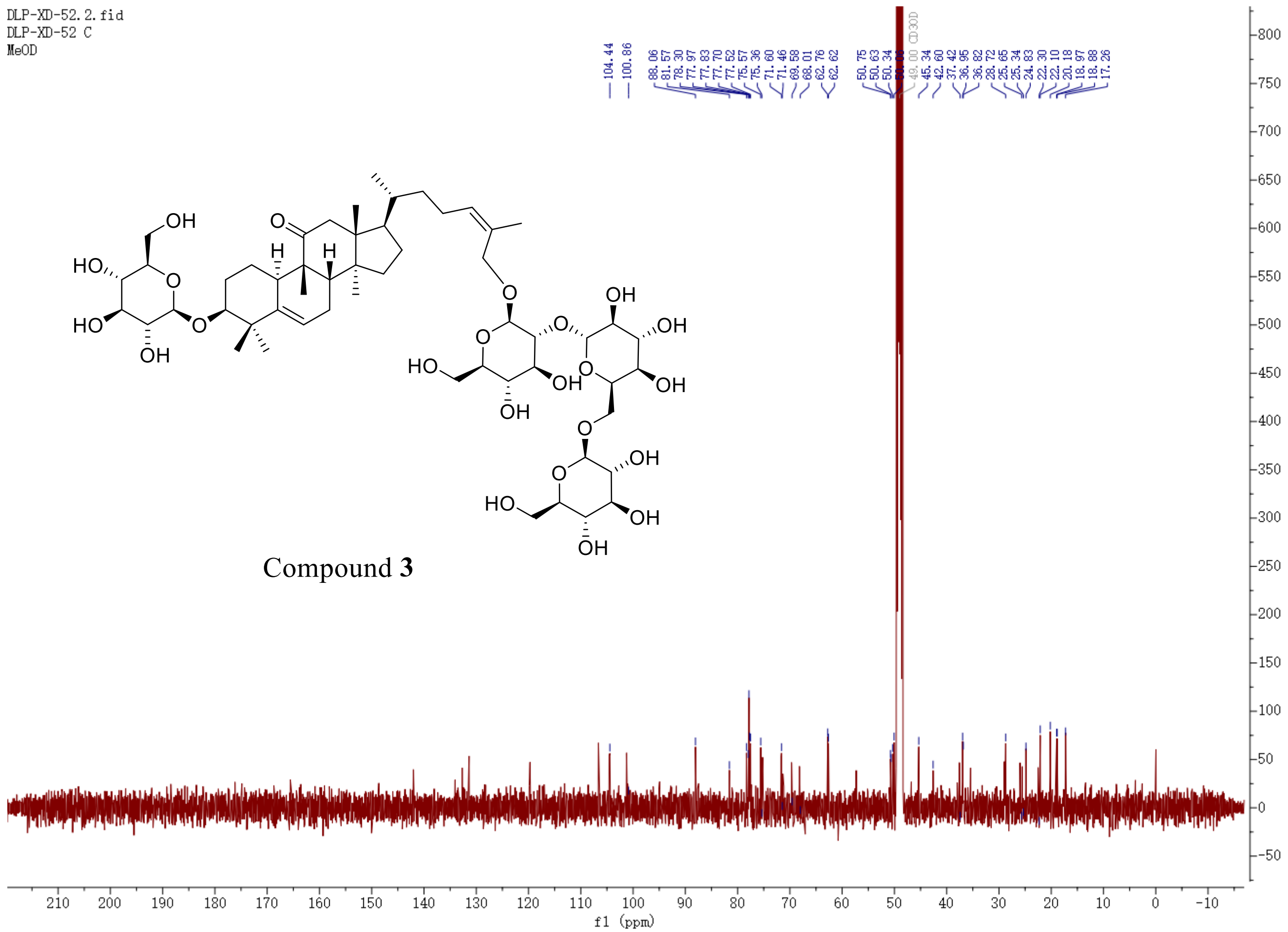

Figure S27. ^{13}CNMR (125 MHz) spectrum of compound **3** in MeOD.

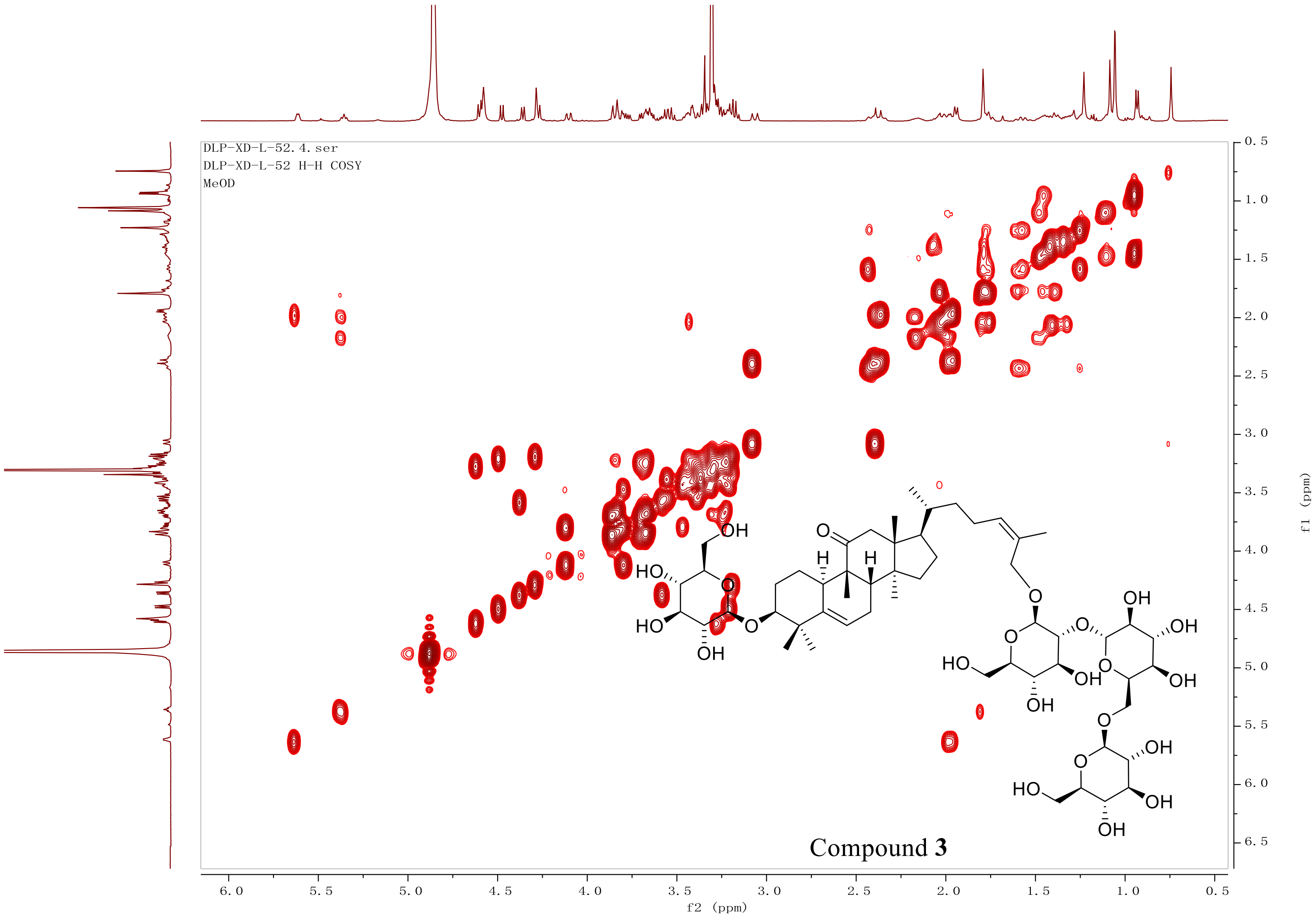

Figure S28. ^{1}H-^{1}H COSY spectrum of compound **3** in MeOD.

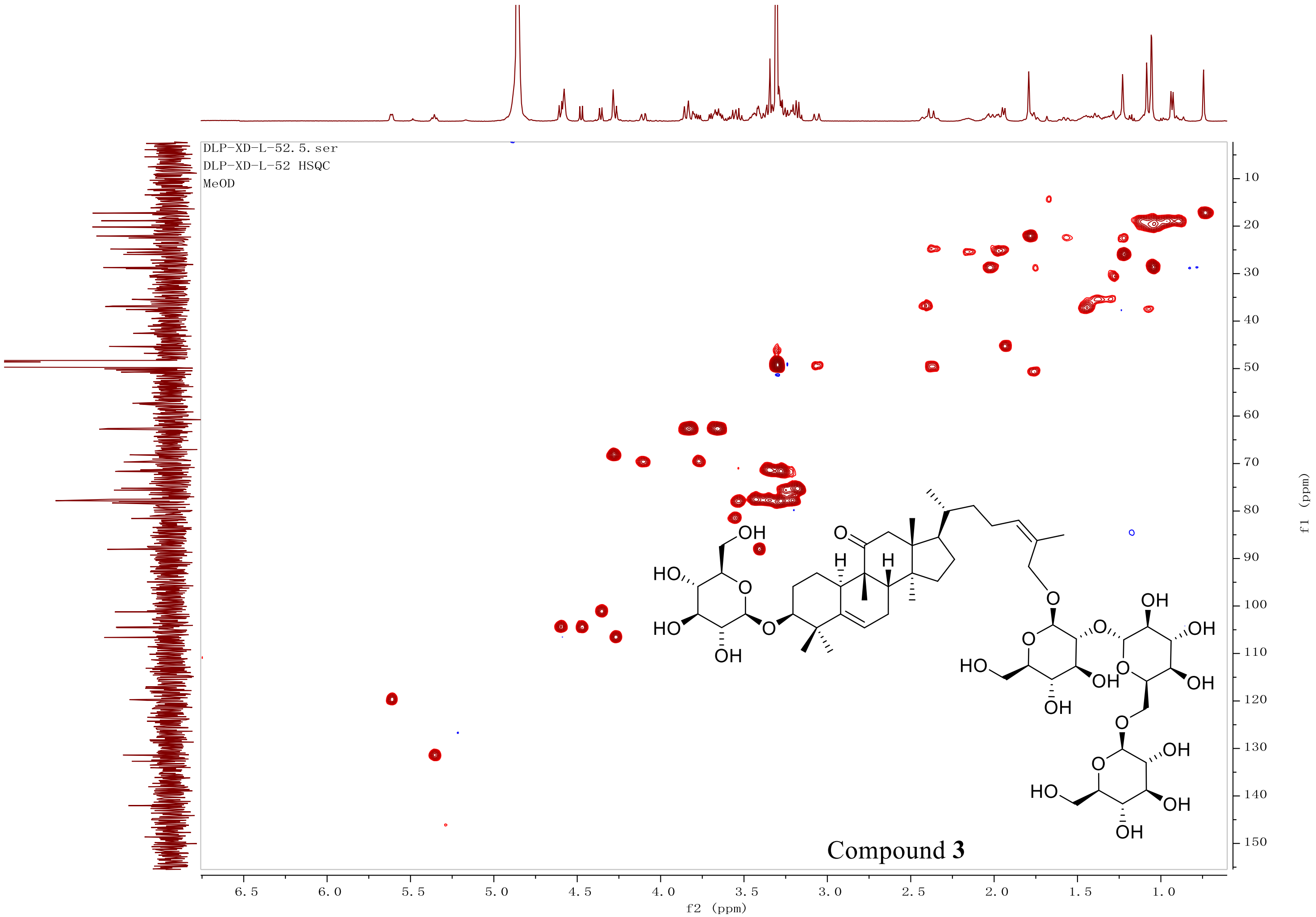

Figure S29. HSQC spectrum of compound **3** in MeOD.

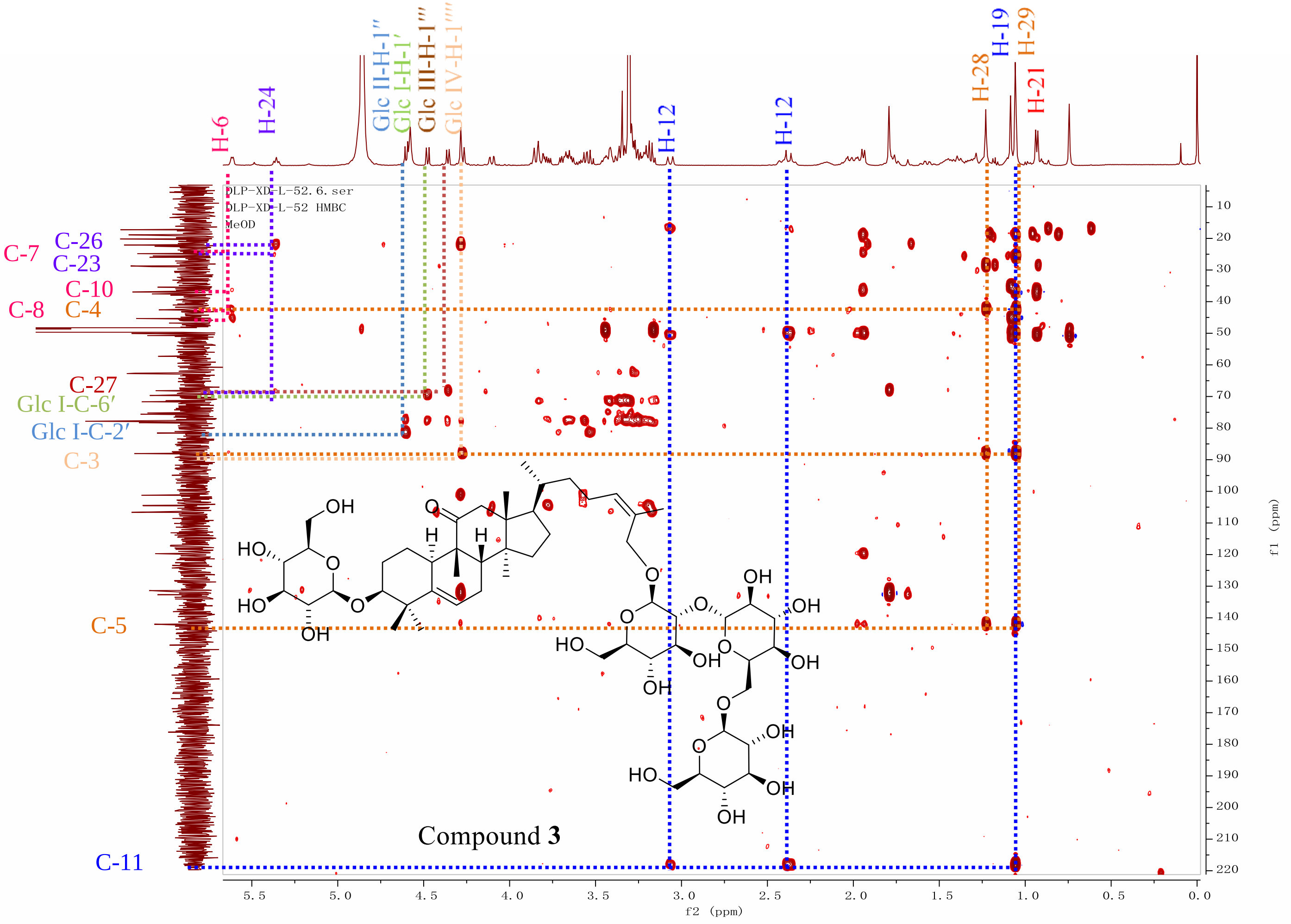

Figure S30. HMBC spectrum of compound **3** in MeOD.

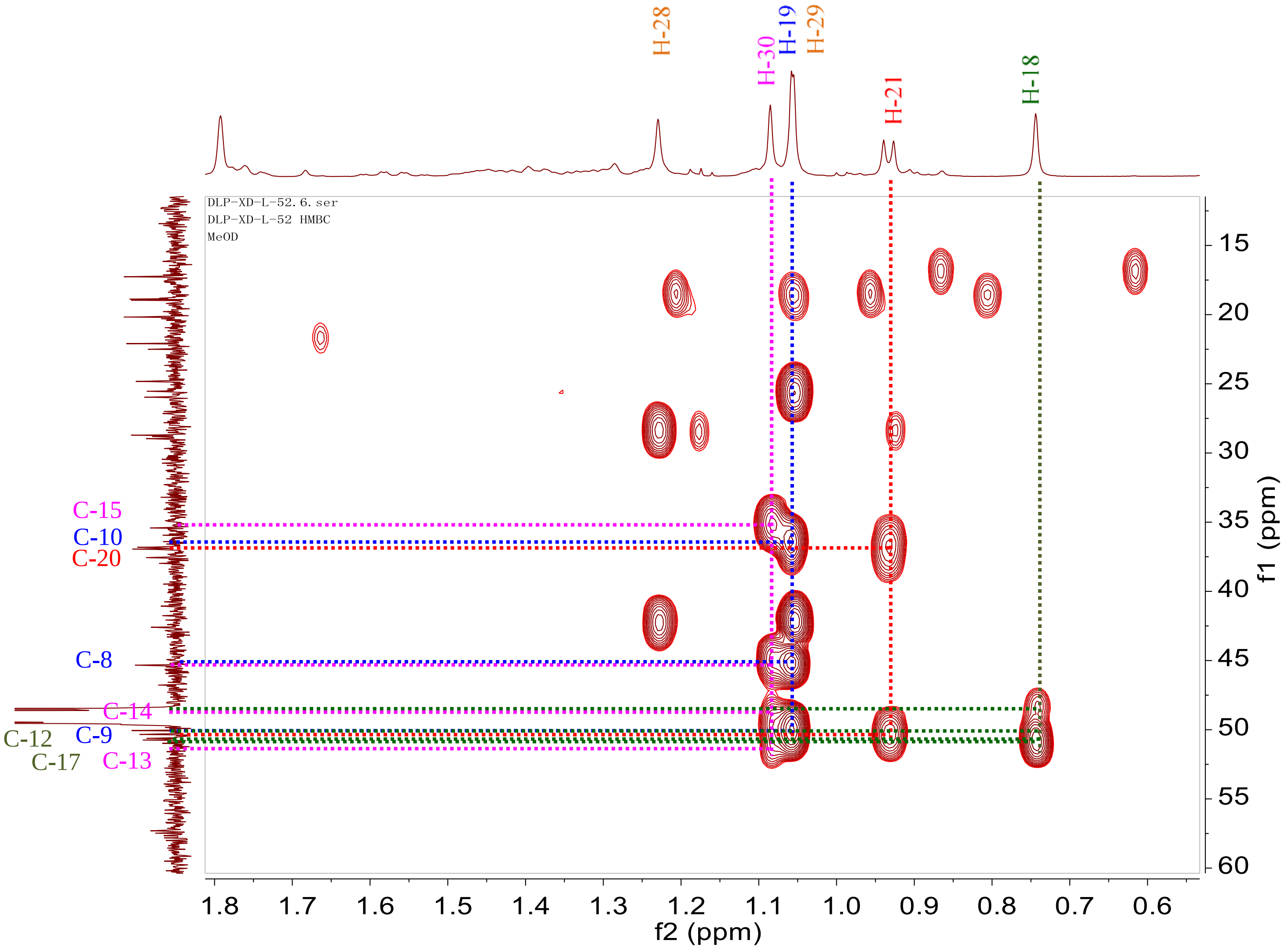

Figure S31. The enlarged HMBC spectrum of compound **3** in 0.6~1.8 ppm in MeOD.

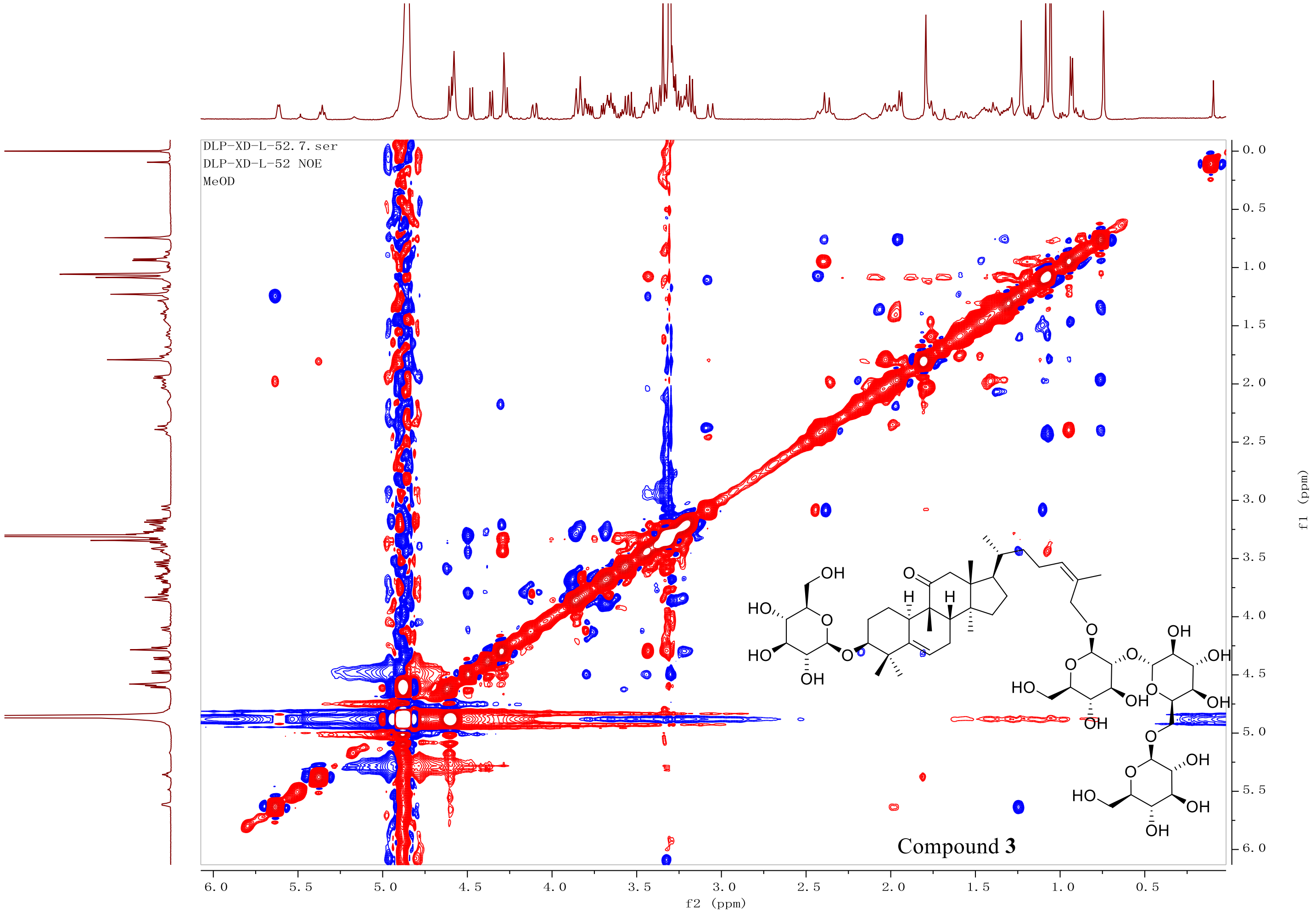

Figure S32. NOESY spectrum of compound **3** in MeOD.

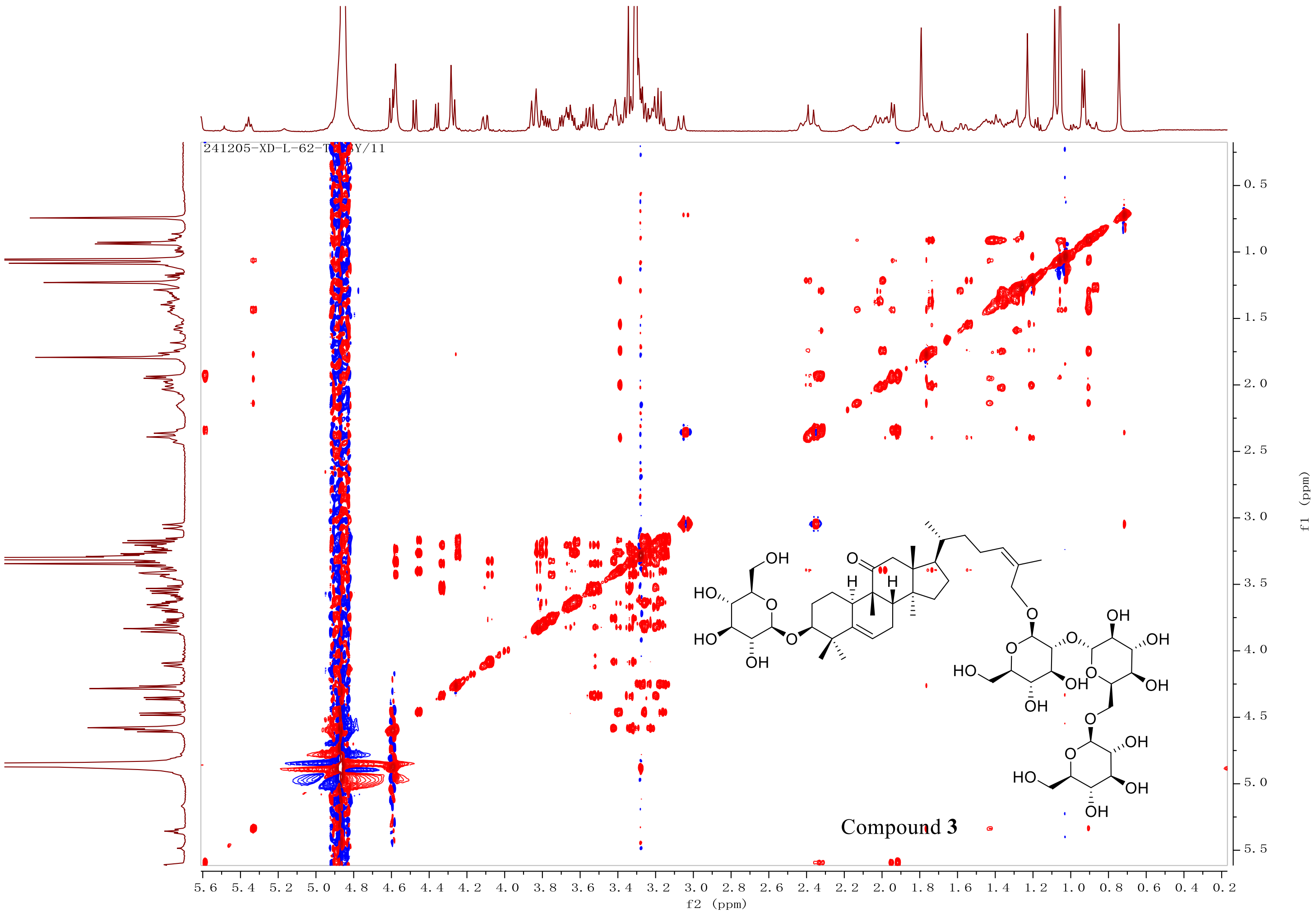

Figure S33. TOCSY spectrum of compound **3** in MeOD.

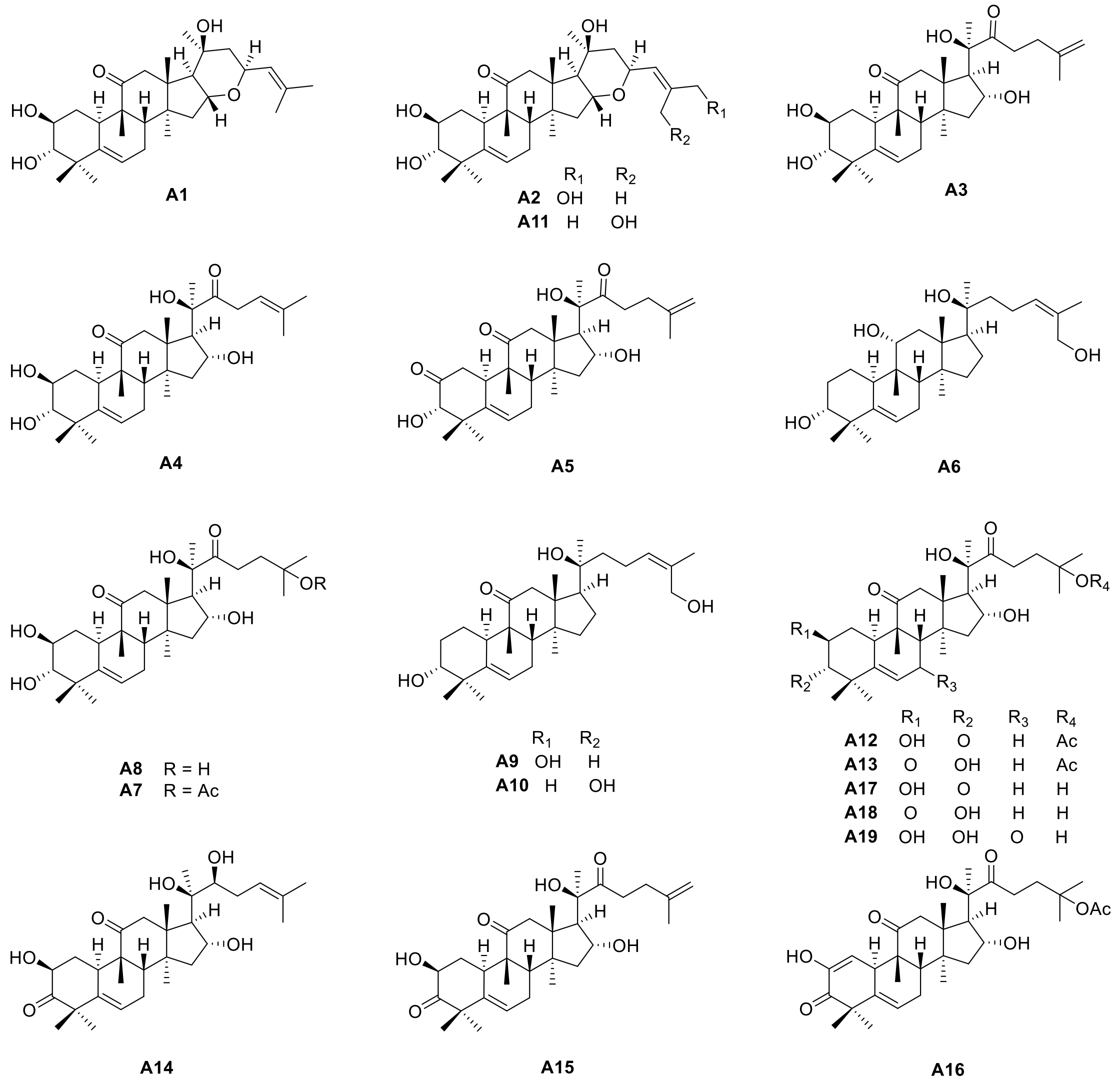

Figure S34. The structures of compounds **A1–A19**.

Table S1. Binding affinity of different compounds to inflammatory pathway related target proteins.

Proteins / Compounds	Binding energy（-kJ/mol）							
	AKT	NLRP3	ASC	caspase-1	Nrf2	OH-1	NF-κB	AMPK
**1**	63.41	46.94	77.97	81.13	85.09	-	106.70	108.54
**2**	67.61	62.09	74.47	92.34	88.94	-	108.86	117.29
**3**	66.23	53.12	85.26	87.52	75.11	98.59	111.37	107.53
**4**	38.68	33.86	43.51	49.15	82.25	63.15	65.44	69.54
**5**	39.83	34.21	49.66	50.92	77.99	61.98	71.29	71.16
**6**	34.21	35.48	47.08	48.66	80.09	60.89	68.60	63.42
**7**	38.12	38.24	54.34	52.19	80.39	60.01	71.69	65.45
**8**	46.81	53.30	69.71	69.01	-	-	106.74	89.62
**9**	57.52	48.41	68.03	80.00	111.24	-	109.37	92.39
**10**	47.35	46.07	57.04	62.74	97.30	-	91.42	84.96
**11**	36.24	29.28	50.42	50.54	71.49	54.30	75.12	61.37
**12**	41.61	32.36	57.30	61.37	89.33	76.56	82.93	72.45
**13**	44.78	41.61	57.77	61.13	87.19	-	89.75	79.35
**14**	50.68	59.85	68.97	71.27	100.02	-	98.70	88.12
**15**	40.06	29.95	56.31	68.17	93.78	53.18	88.58	71.23
**16**	39.55	34.66	52.28	60.33	91.87	-	85.13	76.29
**A1**	24.57	17.45	35.23	37.44	59.00	47.53	50.71	39.80
**A2**	28.89	22.79	35.65	41.68	61.21	49.03	52.25	47.06
**A3**	32.43	24.48	33.62	39.26	65.08	46.92	53.32	49.45
**A4**	31.01	20.85	33.21	40.34	63.50	49.23	52.35	52.25
**A5**	33.24	17.29	36.47	38.95	64.46	50.94	56.32	48.04
**A6**	27.44	23.05	39.04	45.63	58.14	51.49	54.60	46.78
**A7**	33.21	26.16	37.82	49.75	74.40	57.33	64.04	48.97
**A8**	34.53	20.83	39.26	46.14	64.60	44.38	60.02	54.64
**A9**	25.66	20.43	37.70	42.79	58.18	50.42	51.94	50.25
**A10**	28.32	17.70	35.76	45.21	58.54	47.63	52.56	47.03
**A11**	29.25	17.58	42.97	39.39	53.76	48.20	52.50	49.47
**A12**	32.76	22.61	36.84	50.45	71.24	51.89	67.19	59.16
**A13**	41.99	24.05	41.24	50.01	69.78	57.16	57.82	59.90
**A14**	31.60	19.25	35.72	45.36	63.11	48.25	53.22	52.32
**A15**	24.94	18.05	35.52	45.26	63.42	54.02	56.64	50.77
**A16**	30.16	23.92	39.81	42.38	71.00	54.07	62.61	59.69
**A17**	31.12	30.23	44.03	45.40	66.82	50.14	54.17	50.13
**A18**	32.60	22.57	39.58	42.45	67.67	54.77	57.79	53.04
**A19**	35.63	26.11	42.19	45.44	68.93	55.38	58.75	51.68

Table S2. The optimized lowest energy 3D conformers and energy analysis of compounds **1a**–**3a**.

Configurations	Conformers	Structures	E (kJ/mol)	Populations (%)
**2a**	1	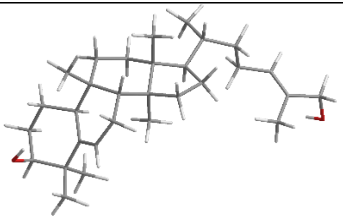	0.00	18.6 %
**2a**	2	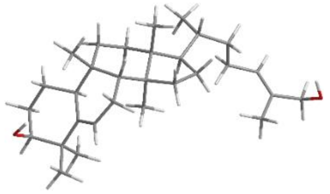	0.27	16.7 %
**2a**	3	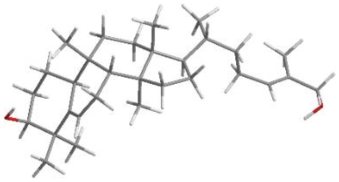	0.63	14.4 %
**2a**	4	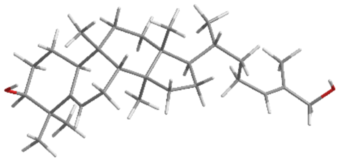	0.96	12.7 %
**3a**	1	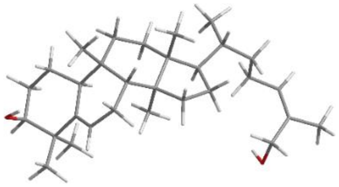	0.00	16.2 %
**3a**	2	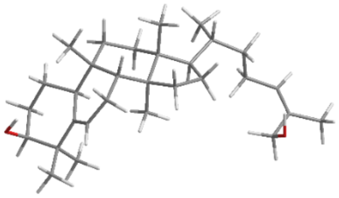	0.24	14.8 %
**3a**	3	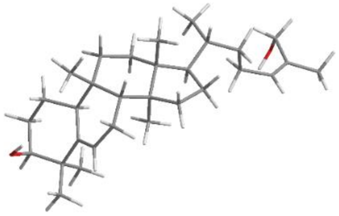	0.72	12.1 %

Configurations	Conformers	Structures	E (kJ/mol)	Populations (%)
**5a**	1	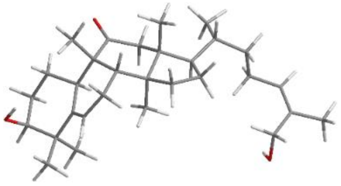	0.00	19.6%
**5a**	2	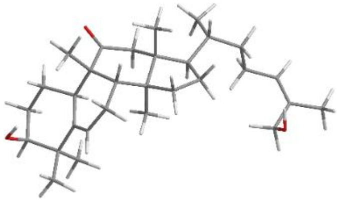	0.23	17.9%
**5a**	3	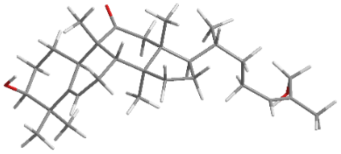	1.04	12.9%
**5a**	4	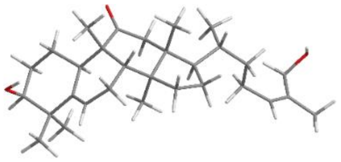	0.63	10.1%
